# AIVT: Inference of turbulent thermal convection from measured 3D velocity data by physics-informed Kolmogorov-Arnold networks

**DOI:** 10.1126/sciadv.ads5236

**Published:** 2025-05-07

**Authors:** Juan Diego Toscano, Theo Käufer, Zhibo Wang, Martin Maxey, Christian Cierpka, George Em Karniadakis

**Affiliations:** ^1^ Division of Applied Mathematics, Brown University, Providence, RI 02912, USA.; ^2^ Institute of Thermodynamics and Fluid Mechanics, Technische Universität Ilmenau, Ilmenau, Germany.; ^3^ Department of Mechanical Engineering, Massachusetts Institute of Technology, Cambridge, MA 02139, USA.; ^4^ School of Engineering, Brown University, Providence, RI 02912, USA.

## Abstract

We propose the artificial intelligence velocimetry-thermometry (AIVT) method to reconstruct a continuous and differentiable representation of the temperature and velocity in turbulent convection from measured three-dimensional (3D) velocity data. AIVT is based on physics-informed Kolmogorov-Arnold networks and trained by optimizing a loss function that minimizes residuals of the velocity data, boundary conditions, and governing equations. We apply AIVT to a set of simultaneously measured 3D temperature and velocity data of Rayleigh-Bénard convection, obtained by combining particle image thermometry and Lagrangian particle tracking. This enables us to directly compare machine learning results to true volumetric, simultaneous temperature and velocity measurements. We demonstrate that AIVT can reconstruct and infer continuous, instantaneous velocity and temperature fields and their gradients from sparse experimental data at a high resolution, providing an additional approach for understanding thermal turbulence.

## Teaser

AIVT infers hidden temperature fields from sparse turbulent velocity data using physics-informed cKANs.

## INTRODUCTION

Turbulence is considered the last frontier in classical physics. Thermal processes predominantly drive turbulent convection at both terrestrial and astrophysical scales (*1*–*4*), leading to the joint transport of heat, mass, and momentum. Beyond its natural occurrence, thermal convection also plays a crucial role in many engineering and technological applications (*5*–*7*). Therefore, understanding the underlying mechanisms is crucial for gaining better insights into our environment and ensuring the success of engineering applications, such as the transition from fossil fuels to renewable energy (*8*).

Since Bénard (*9*) and Rayleigh (*10*) pioneered the study of thermal convection, researchers have extensively studied this phenomenon using both experimental and computational methods (*11*–*13*). Although increasingly powerful computers have substantially enhanced our ability to study thermal convection and turbulent flows, in general, through direct numerical simulation (DNS) (*14*, *15*), the immense computational cost limits these simulations to research problems, simple geometries, and moderate Reynolds numbers.

In contrast to simulations, which rely on assumptions and simplifications, experimental methods inherently reveal the physics of the system, allowing insights into systems that cannot accurately be modeled with numerical simulations. Although flow measurement techniques have advanced substantially in recent decades (*16*, *17*), these methods often suffer from limited spatiotemporal resolution, restricted coverage, and method-specific uncertainties for turbulent flows. Additionally, the complexity increases markedly when simultaneously measuring different quantities, such as temperature and velocity in thermal convection. Combined volumetric temperature and velocity measurements have been reported in very few studies (*18*–*24*). Out of these studies, only Käufer and Cierpka (*24*) measured temperature and velocity in turbulent thermal convection simultaneously at the same point.

Because temperature measurements often account for most of the complexity (*24*), several approaches combine numerical methods with experimental observations to derive temperature information from velocity data. For instance, Noto *et al.* (*25*) used the energy equation to estimate the mean temperature field from PIV velocity data and boundary conditions for a quasi–steady-state flow in a quasi–two-dimensional (2D) Hele-Shaw cell. Similarly relying on traditional approaches, Bauer *et al.* (*26*) attempted to reconstruct the temperature in Rayleigh-Bénard Convection (RBC) from tomographic PIV measurements using DNS, but their validation was limited to mean temperature fields in small regions due to the restrictions and quality of the experimental data.

Recently, deep learning (DL) has emerged as an alternative for studying turbulent thermal convection (*27*–*29*). In particular, Teutsch *et al.* (*27*) used a U-net to predict temperature from planar stereoscopic particle image velocimetry data. However, the black-box nature of traditional DL approaches introduces a lack of interpretability, requires extensive data, and limits reconstruction capabilities to the information encoded in the training dataset, preventing the inference of hidden fields.

Physics-informed machine learning (PIML) overcomes the limitations of both DL and traditional methods by encoding the regularization of the governing equations into a flexible representation model. The PIML approach uses a multilayer perceptron (MLP) or a Kolmogorov-Arnold network (KAN) to approximate the solution of ordinary differential equation/partial differential equation (PDE) by minimizing a loss function that fits observable data while satisfying the underlying physical laws (*30*, *31*). Since the introduction of PIML (*30*), researchers have focused on further increasing its performance through various means, which can be roughly classified into three categories: representation model modifications (*32*–*37*), PDE reformulations (*38*–*40*), and optimization algorithm enhancements (*35*, *41*–*47*).

PIML’s flexibility makes it optimal for inverse problems, as it allows researchers to obtain solutions with partial or no knowledge of boundary conditions, which typically limits the applicability of traditional numerical methods. Consequently, researchers have extended the PIML framework to study heat transfer (*48*–*51*), thermal convection (*52*, *53*), and turbulence (*54*–*56*). Although most of these studies have been limited to synthetic data and idealized simulations (*57*), they have revealed the vast potential of PIML capabilities to use incomplete data in complex environments. For instance, by reconstructing flow fields from easily accessible data (e.g., concentration, temperature, and 2D velocities), PIML can infer hard-to-access quantities [e.g., 3D velocities (*58*), wall-shear stress (*59*), or pressure gradients (*60*)], without requiring ground-truth data, which is not viable with traditional DL methods. These capabilities have primarily been explored and introduced under artificial intelligence velocimetry (AIV) (*59*, *60*) or related approaches (*58*). However, these studies were limited to laminar flows or idealized scenarios. Inferring the temperature from measured 3D velocity data by PIML has not yet been successfully demonstrated for the following main reasons:

1) The complexity of PIML optimization arises from minimizing a multi-objective loss function, which becomes particularly challenging when dealing with highly nonlinear, multi-scale, chaotic behavior or noisy experimental data (*41*), typical of turbulent thermal convection.

2) The lack of reliable volumetric experimental temperature and velocity data for training and validation purposes. Real-world turbulence is inherently 3D, requiring the solution of gradients in all three spatial dimensions and time to solve the PDEs accurately.

To address these challenges, we present artificial intelligence velocimetry-thermometry (AIVT). This approach extends the AIV framework (*59*, *60*) to infer hidden turbulent temperature fields and reconstruct the velocity field from 3D Lagrangian velocity measurements.

First, to address the complexity of PIML optimization, we introduce an innovative component: residual-based attention with resampling (RBA-R), which dynamically rescales and resamples collocation points by focusing on high-error regions, thereby promoting uniform error minimization. Moreover, we reformulate the PDEs into their vorticity formulations, eliminating the dependence on pressure and allowing us to infer temperature purely from velocity observations. Our study details how these enhancements, combined with a strategic sequential training protocol that progresses from simple to complex features, enable effective processing and learning of turbulent flow fields. Additionally, to enhance our model’s representation capabilities, we leverage the recently proposed Chebyshev Kolmogorov-Arnold networks (cKANs) (*31*), which have empirically demonstrated robustness to noise and, in certain cases, despite using fewer parameters, exhibit accelerated convergence compared to standard MLPs (*61*). To further understand cKAN performance, we formally prove that this representation model can be regarded as an MLP whose inputs, intermediate features, and outputs have been enriched using a Chebyshev expansion. This structured enrichment induces sparse orthogonality, which may explain why cKANs tend to require fewer parameters. These methodological advancements offer a comprehensive solution that bridges gaps in the current state of scientific machine learning, particularly in turbulence and related fields, where traditional PIML approaches often fall short.

Second, we further refine the data processing and extend the work of Käufer and Cierpka (*24*) to obtain a set of simultaneously measured 3D temperature and velocity data. This dataset allows us to validate our model using temporally and spatially resolved 3D temperature and velocity measurements. We compare the inferred temperature, heat transfer, and reconstructed velocity directly with the measurement data. Furthermore, we demonstrate the capabilities of AIVT to estimate gradients of the temperature and velocity fields and compute related quantities such as vorticities, distributions of invariants of the velocity gradient tensor, and thermal and viscous dissipation rates, which we extensively compare to the literature.

Our findings suggest that combining reliable experimental data with scientific machine learning can substantially advance fluid mechanics research by leveraging the strengths of both measurements and scientific machine learning while overcoming their individual limitations.

## RESULTS

### Problem description

**Fig. 1.**
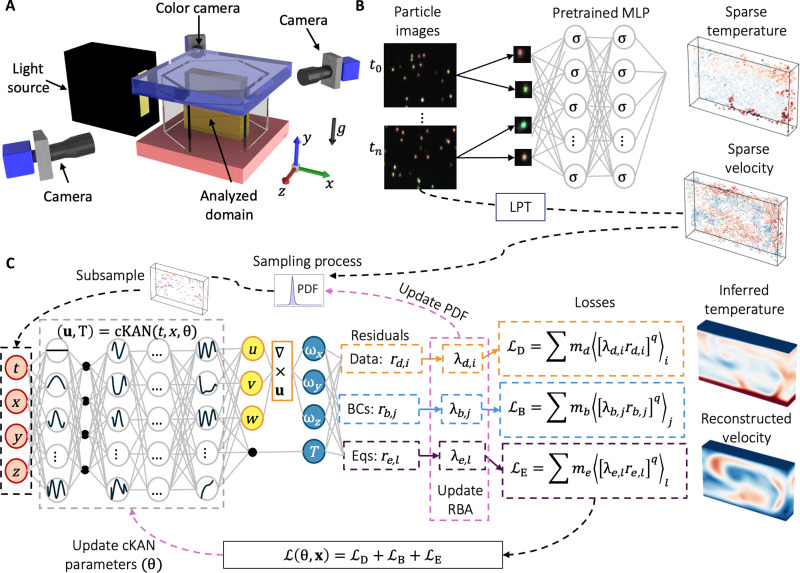
Problem setup. (**A**) The experimental setup includes a hexagonal RBC cell, cameras, and a light source. The illuminated vertical slice appears in yellow. Gravity *g* (black arrow) acts in a negative y direction. We conducted the experiment at $\text{Ra}=3.4\times 10^{7}$ and *Pr* = 10.6. (**B**) This schematic illustrates the joint temperature and velocity measurement process. We use the Shake-the-Box method (DAVIS 10.2, LaVision GmbH) to obtain particle positions and velocity vectors. We derive particle temperatures from color images using a MLP pretrained on calibration data. (**C**) During each training iteration, we sample the domain on the basis of a probability density function (PDF) that identifies high-error regions. We feed these coordinates into a modified Kolmogorov Arnold network (cKAN) to predict 3D velocities and temperature. We calculate residuals for data, boundary conditions, and equations, with derivatives obtained via automatic differentiation. Residual-based attention (RBA) scales these residuals, balancing contributions from each training point, and we update cKAN parameters to yield continuous temperature and velocity fields.

We obtain the experimental data by combining particle image thermometry (PIT) (*62*) and Lagrangian particle tracking (LPT) (*63*). In particular, we use thermochromic liquid crystals (TLCs) as tracer particles to study Rayleigh-Bénard convection at Rayleigh number (*Ra*) = 3.4 × 10^7^ and Prandtl number (*Pr*) = 10.6 in a hexagonal container. As shown in Fig. 1A, we studied a vertical slice at the cell center using two monochrome cameras and one color camera. The recorded images were processed by LPT, which provides 3D particle positions and velocities along particle trajectories (see Fig. 1B). To determine the temperature information, we feed the individual color particle images into a pretrained MLP that estimates the particle temperature from the corresponding particle’s color image. Further details on the setup and the proposed framework can be found in Materials and Methods and (*24*). We improve the referenced approach by applying a lightweight MLP and a more strict $L_{2}$ regularization, improving the generalization of the model, especially for temperatures in between the discrete calibration data points. Furthermore, we now enforce a closer match between the back-projected and detected particle positions and center the extracted particle image around its highest intensity peak, resulting in fewer but even more reliable data points. As a result, we obtained about 1000 snapshots with ~3000 Lagrangian velocity and temperature data points per snapshot, of which we used 282 snapshots corresponding to 20 nondimensional times. Further details on the setup and the proposed framework can be found in Materials and Methods and (*24*).

On the basis of these experimental data and the flexibility of PIML to incorporate the underlying physical laws (*57*, *59*, *60*), we introduce AIVT. AIVT is a scientific machine learning method based on physics-informed cKANs (cPIKANs) (*31*) and AIV (*59*, *60*) that uses the full set of Navier-Stokes equations including the energy equation to model turbulent thermal convection (*13*). As shown in Fig. 1C, we use AIVT to infer 3D continuous and differentiable velocity [u=(u,v,w)] and temperature (T) fields from the discrete experimental observations of velocity. We train our model by optimizing a combined loss function (L) that minimizes the residuals (i.e., point-wise error) of the velocity data $\left(r_{d}\right)$, boundary conditions $\left(r_{b}\right)$, and governing equations $\left(r_{e}\right)$.

To deal with the local imbalances related to optimizing L, we introduce RBA-R. RBA-R uses residual-based attention (RBA) (*41*) weights ($\lambda_{i}$) as local multipliers to balance the point-wise errors, enabling a uniform convergence along the analyzed domain (*64*). Additionally, because $\lambda_{i,d}$, $\lambda_{i,b}$, and $\lambda_{i,e}$ contain historical information of the residuals, we use them to compute for each loss component ($L_{B}$, $L_{D}$, and $L_{E}$) an RBA-based probability density function (PDF) used to resample the high-error regions. To ensure the exact enforcement of the constraints, we follow Sukumar and Srivastava (*33*) and use approximate distance functions (ADFs) to impose the temperature boundary conditions and redesign the base KAN model to infer divergence-free fields.

Last, to simplify the inherent optimization problem related to training a multi-objective loss function, we propose a sequential learning approach. In the first step, we solve a purely data-driven problem, where the model mainly fits the data and the boundary conditions. Subsequently, we train the model using a lower *Ra* (i.e., “partial physics”), which enables capturing the diffusive features of the desired solution. The first steps can be considered as a “guided” initialization that prepares the model to learn the desired temperature and velocity fields at higher Ra. The specific details of this method—the corresponding ablation study with their respective loss landscapes, a comprehensive comparison between MLPs and cKANs, and a formal proof of their relationship—are detailed in the Supplementary Materials.

We train our AIVT model with 50% of the experimental velocity measurements in the core region (0.1 < *y* < 0.9) and validate our results with the remaining unseen data. Because our model outputs are continuous and differentiable, we derive additional 3D fields such as convective heat transfer, vorticity, and viscous and thermal dissipation rates that are hard to measure or not even directly measurable. Additionally, we compute the turbulent flow statistics, including mean fields, root-mean-squared fluctuations, viscous and thermal dissipation rates, and the Q and R invariants of the velocity gradient tensor. All quantities are obtained in dimensionless form based on the characteristic units [see (*1*)]. We compare the results with the available measurement data and values from the literature and find good agreement.

### Reconstructed velocity

To assess the capability of the AIVT method to reconstruct the velocity field, we compare the model predictions on the validation dataset (i.e., 50% of the measured velocity data). The average relative $L_{2}$ errors (see eq. S30) on the core region (0.1 < *y* < 0.9), for u, v, and w, are 9.6, 10.8, and 11.9%, respectively, and the uncertainty due to the model parameters is less than 0.25%. The details per velocity component are shown in fig. S7.

**Fig. 2.**
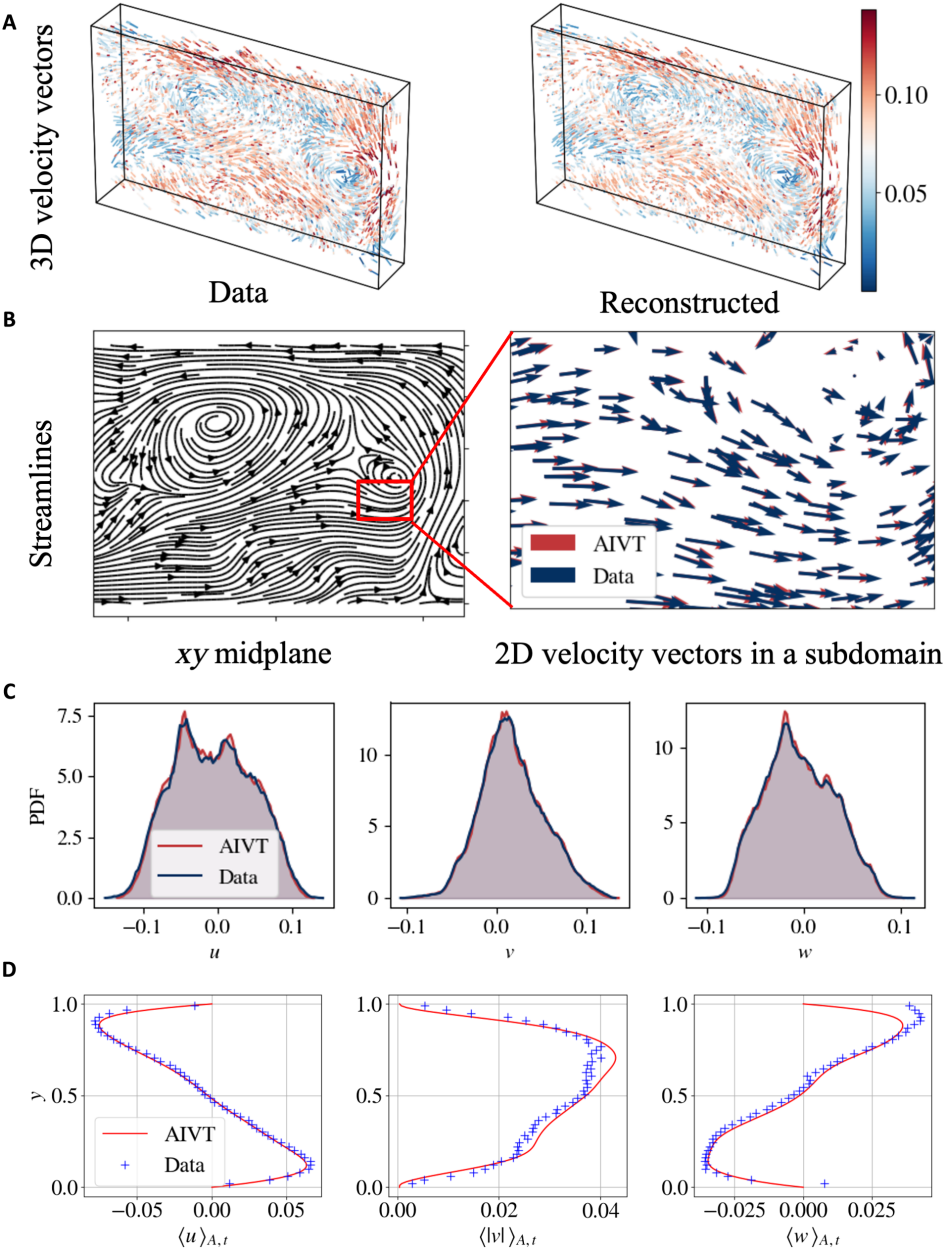
Velocity reconstruction. (**A**) Exemplary instantaneous velocity vectors of the measured and reconstructed velocity at the particle positions in 3D space. The velocity magnitude is color coded. The point-wise comparison of each velocity component for the remaining time steps is shown in movie S1. (**B**) Streamline plot of the u and v velocity in the xy plane at z=0 and detailed view of the measured (blue) and inferred velocity (red). (**C**) PDFs of the measured (blue) and reconstructed (red) velocity components at particle locations. (**D**) Vertical profile of mean velocity fields in the x (${\langle u\rangle }_{A,t}$), y (${\langle |v|\rangle }_{A,t}$), and z (${\langle w\rangle }_{A,t}$) directions. On the basis of the profiles of horizontal velocity components, the orientation and rotation direction of the LSC can be determined.

Figure 2A shows the 3D vector plot of the measured and reconstructed velocities at particle locations for an exemplary snapshot. When comparing the plots, we see that measured and reconstructed velocities are almost indistinguishable. Figure 2B shows the streamlines from velocity components in the x and y directions, u and v, in the xy plane. The streamline plot indicates the presence of the well-known large-scale circulation (LSC) as it is typical for thermal convection with aspect ratios close to unity (*65*). To analyze the difference between the prediction and measured data, we show a detailed comparison of the measured (blue) and the reconstructed (red) velocity vectors in a 2D subdomain. The detailed view of the velocity vectors confirms that the measured and reconstructed velocities match closely. To obtain a global measure of the reconstruction quality, we compare the PDFs (see Fig. 2C) of the three velocity components obtained from the measurements (blue) and reconstruction (red). For all velocity components, we observe that PDFs of the measurements and reconstructions align.

To compare the measured and reconstructed mean velocity fields, Fig. 2D shows the averaged vertical velocity profiles ${\langle u\rangle }_{A,t}$, ${\langle |v|\rangle }_{A,t}$, and ${\langle w\rangle }_{A,t}$ (red) and the respective binned and averaged measured velocity components (blue crosses). Note that, unlike the PDFs of the velocity, we compare the high-resolution reconstructed velocity with the sparse binned velocity measurements. Comparing the profiles, we observe that the reconstructed and measured ${\langle u\rangle }_{A,t}$ profiles collapse. Both profiles show the highest and lowest velocity at *y* ≈ 0.1 and *y* ≈ 0.9, respectively, with zero or close to zero mean ${\langle u\rangle }_{A,t}$ at *y* = {0, 0.5, 1}. The shape of the profile is representative of the LSC. Also, the profiles of ${\langle |v|\rangle }_{A,t}$ align; both profiles indicate the highest velocity magnitude at *y* ≈ 0.7. The limited volume of investigation only partially captures the LSC, which causes the asymmetry of the profiles with respect to *y* = 0.5. The profiles of ${\langle w\rangle }_{A,t}$ show a similar shape to those of ${\langle u\rangle }_{A,t}$ albeit with a flipped sign and at half the magnitude. From the combined information of the ${\langle u\rangle }_{A,t}$ and ${\langle w\rangle }_{A,t}$ profiles, we can determine the orientation and rotation direction of the LSC. Comparing the measured and reconstructed profiles, we observe that both profiles coincide with the region close to the top plate. In this region, ${\langle w\rangle }_{A,t}$ is overestimated by the measurements. Due to the limited depth of the domain and the camera positioning, the determination of the z position is the most difficult one. Furthermore, particles close to the cooling plate are far below the sensitivity range of the TLCs and, thus, appear almost transparent. The comparison, however, shows that the AIVT model can successfully reconstruct velocities in regions hard to access by measurements (typically close to walls) and validates our previous observation of closely matching reconstruction results statistically.

**Fig. 3.**
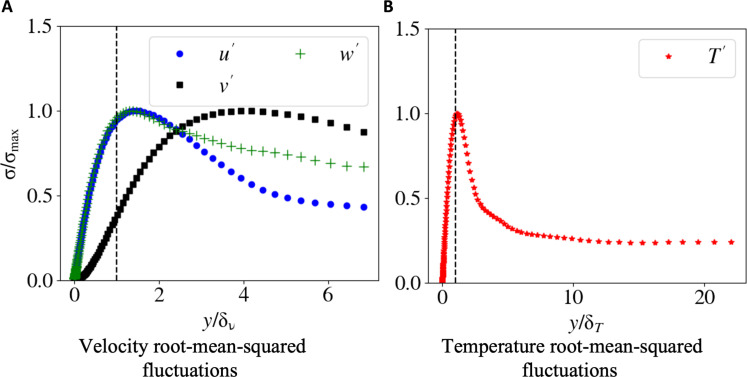
Velocity and temperature fluctuations profiles. (**A**) Normalized root-mean-squared [$\sigma /{\left(\sigma \right)}_{\text{max}}$] velocity u′,v′, and w′ and (**B**) temperature T′ fluctuations on the top half domain. The vertical dashed lines indicate the viscous $\delta_{\nu }$ and thermal $\delta_{T}$ boundary layer thicknesses based on (*9*) and (*8*), respectively. The profiles are consistent with results reported by (*66*, *74*, *75*) and scaling theory (*73*, *76*).

To further investigate the velocity reconstruction performance of the AIVT model in the top boundary region, we show the normalized root-mean-squared [$\sigma /{\left(\sigma \right)}_{\text{max}}$] profiles of the velocity fluctuations, defined in Eqs. 4 to 6, plotted over multiples of the viscous boundary layer thickness $\delta_{\nu }$ in Fig. 3. The dashed line indicates the estimated viscous boundary layer thickness according to Eq. 9. The profiles for the horizontal velocity fluctuations u′ (blue) and w′ (green) are virtually identical up to approximately two viscous boundary layers from the wall and show the typical profile as reported in (*66*). Furthermore, the position of the profile maxima, which can be used to estimate boundary layer thickness (*67*), is close to the boundary layer thickness derived from scaling theory. The plate-normal or vertical fluctuation profile v′ (black) differs from the horizontal fluctuation profiles due to the absence of a main shear flow necessary for a Blasius-type boundary layer to emerge. The Blasius-type boundary layer has been found to be a suitable model for RBC convection with a dominant LSC (*68*).

### Inferred temperature and convective heat transfer

As outlined in the problem description, AIVT’s main advantage is that it estimates hard-to-measure quantities like temperature from more easily experimentally accessible quantities like velocity. To validate our model’s temperature predictions, we use all the available experimental data and obtain an average relative $L_{2}$ error of 3.62% with an uncertainty of less than 1% (see table S1).

**Fig. 4.**
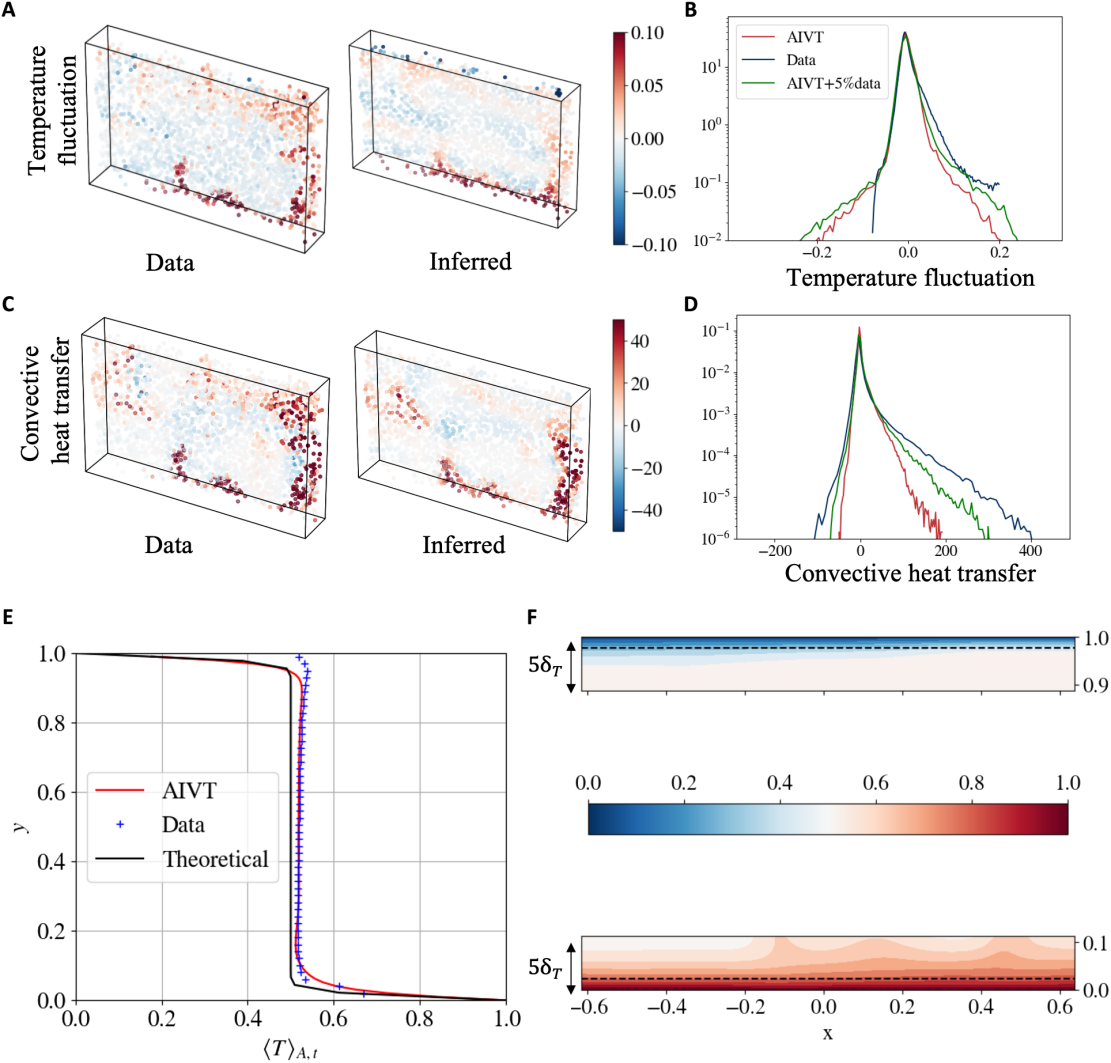
Inferred temperature and heat transfer results. (**A**) Scatter plot comparison of measured and inferred temperature fluctuations at particle positions for a representative snapshot. (**B**) PDFs of measured (blue), inferred (red), and reconstructed (green) temperature fluctuations at particle locations. The TLCs’ nonuniform sensitivity shifts the measurable temperature range toward positive values. (**C**) Scatter plot comparison of measured and inferred convective heat transfer *J* at particle positions for a representative snapshot. (**D**) PDFs of measured (blue), inferred (red), and reconstructed (green) convective heat transfer J at particle locations. Measurement uncertainties extend the PDF tails, leading to wider distributions, while cPIKANs produce smoother results. Including a few temperature observations during training improves the agreement with measurements. (**E**) Vertical profile of mean temperature ${\langle T\rangle }_{A,t}$. The red line shows the inferred profile, blue markers represent binned Lagrangian data, and the black line shows the theoretical profile from (*70*). (**F**) Temperature field view at *z* = 0 in the top and bottom boundary regions, covering 5 $\delta_{T}$ from each plate. The black dashed line indicates the thermal boundary layer thickness, $\delta_{T}$. Notably, despite being trained on a limited number of collocation points, the model successfully reconstructs a substantially higher-resolution field, capturing fine-scale boundary layer structures.

In Fig. 4A, we compare exemplary snapshots of the measured and inferred temperature fluctuation Θ (see Eq. 2) at the particle positions. The direct comparison shows that the AIVT approach correctly infers the detaching thermal plumes and temperature structures. However, the thermal plumes appear more pronounced in the scatter plot of the measured temperature fluctuations.

To analyze the inference quality statistically, we computed the PDFs of the measured (blue) and inferred (red) temperature fluctuations, which are shown in Fig. 4B. This plot additionally features the PDF of the temperature fluctuations reconstructed by the AIVT model when trained on 5% of the velocity and temperature data (green). Most noticeably, the PDF of the measured temperature fluctuation is not symmetric around zero as expected in RBC within the Boussinesq approximation regime but shifted toward higher values. This is related to the nonuniform temperature sensitivity and limited range of the TLCs. Comparing the PDFs of the measured and inferred temperature fluctuations, we see that they match for values close to zero but deviate toward the tails. On the one hand, this might be related to the bias of the measurement technique toward higher values and measurement uncertainties that spread the PDFs. On the other hand, this could also be caused by a slight smoothing of the AIVT model. This is further supported by considering the PDF of the reconstructed temperature fluctuations, whose positive tail is more closely aligned with the measured temperature fluctuations, suggesting that, by feeding only a few temperature data points, the smoothing of the AIVT model can be reduced.

Because the joint measurement of temperature and velocity enables us to calculate the convective heat transfer directly, we present snapshots of the measured and inferred convective heat transfer at the particle positions in Fig. 4C. The scatter plots show the same time instance as the scatter plot of the temperature fluctuations. Looking at the scatter plots, we observe the increased convective heat transfer associated with the thermal plumes. When comparing the two scatter plots, we see the strong similarity in the structures even though the high convective heat transfer region at the top right of the measured results is not as pronounced in the inferred results. In both cases, regions of negative local heat transfer are visible. While in RBC, generally positive temperature fluctuations and positive vertical velocity as well as cold temperature fluctuations and negative vertical velocity are correlated, there are regions where, due to viscous forces, adjacent colder fluid is dragged upward or hot fluid downward, respectively (*69*). This shows that the AIVT model is capable of inferring these regions.

Similar to the temperature, we present the PDFs of the convective heat transfer in Fig. 4D to obtain an overall measure of the inference quality. The plot shows the PDFs of the measured (red), inferred (blue), and reconstructed (green) heat transfer. We observe that all PDFs are skewed toward positive values because overall heat is transferred from the bottom to the top of the system. The PDF of the inferred convective heat transfer shows the highest probability for small-magnitude convective heat transfer events. Besides that, the curves match closely in the vicinity of J=0 but differ regarding the positive and negative tails. Here, we see that the PDF of the measured convective heat transfer shows the widest spread, followed by the PDF of the reconstructed convective heat transfer, while the inferred heat transfer shows the least spread. This is consistent with our observations from the PDFs of the temperature fluctuations and the scatter plot of the convective heat transfer. Again, we observe that the inferred AIVT results are smoothed compared to the measurement data and that by including a few temperature observations, the reconstructed convective heat transfer matches the experimental observation more closely.

Another important aspect is the vertical profile of the temperature ${\langle T\rangle }_{A,t}$ averaged over time and along the horizontal directions. Therefore, in Fig. 4E, we compare the inferred vertical temperature profile (red), the temperature profile from the binned measurement data (blue crosses), and the theoretical temperature profile (black) as proposed by Shishkina and Thess (*70*). By contrasting the profiles, we observe a close match between the binned measurements and the inferred temperature profile in the bulk region. In comparison to the theoretical temperature profile, the measured and inferred temperature profiles show slightly higher temperatures in the bulk region. Because this trend is prevalent in the measured and inferred temperature and the shift toward higher temperatures agrees qualitatively with numerical results for non-Boussineq RBC in pure water by Horn *et al.* (*71*), we attribute this to a minor deviation of the experiment from the idealized Boussinesq approximation model.

The discrepancies between the binned measurements and the inferred and theoretical temperature profiles in the boundary region are related to the sensitive range of the TLCs. Adjusting the observation angle reduced the measurable temperature range but was required to achieve sufficient temperature resolution to resolve the temperature fluctuations in the bulk. Thus, very high and low temperatures, as they occur in the regions close to the plates, cannot be measured. This is an inherent trade-off of the applied measurement technique. The effect is more pronounced for low temperatures because the temperature range is not exactly centered around the mean temperature but extends further toward higher temperatures. The reason is that the TLCs show the lowest uncertainty at lower temperatures, and the measurements aimed to resolve small fluctuations around the mean temperature as accurately as possible. The AIVT model is able to infer the temperature close to the wall and the inferred and theoretical temperature profile collapse in the cold boundary region. For the hot boundary region, the inferred temperature profile shows a less steep gradient but follows the data. Hence, the deviation from the theoretical profile may be caused by the constrained region of interest and time span, as well as the presence of detaching thermal plumes, which have been shown to extend the thermal boundary layer locally (*72*).

One of the main advantages of PIML models is that the predictions can be evaluated at any arbitrary point inside the domain. Thus, we use the AIVT model to infer the temperature in the boundary regions that cannot be visualized from the experimental observations. Figure 4F shows the temperature in the top and bottom boundary regions up to five thermal boundary layer thicknesses $\delta_{T}$ in the *xy* plane at *z* = 0. The thermal boundary layer thickness is estimated according to Eq. 8 with the Nusselt number (Nu)=22 being estimated based on the Grossmann-Lohse theory (*73*) and is indicated by the horizontal black dashed line. In the hot boundary region, we observe the presence of steep temperature gradients beyond the indicated theoretical boundary layer thickness and the footprint of the detaching hot thermal plumes, which are visible in the scatter plots of the temperature and convective heat transfer. This is consistent with the results by Tegze and Podmaniczky (*72*), who showed that detaching thermal plumes locally extend the thermal boundary layer further into the bulk. For the cold boundary region, we see a close match of the steep temperature gradient with the boundary layer thickness.

To further analyze the thermal boundary layer, we show the normalized root-mean-squared [$\sigma /{\left(\sigma \right)}_{\text{max}}$] temperature fluctuation profile plotted over multiples of the thermal boundary layer thickness in Fig. 3B. The profile shows the distinct shape also reported by others (*74*, *75*). The position of the profile’s maximum, which is commonly used to estimate the boundary layer thickness (*67*), almost perfectly matches the thermal boundary layer thickness estimated from scaling laws (*73*, *76*), showing that inferred temperature results are consistent with well-established and proven theory. These results demonstrate that the AIVT model is capable of inferring the temperature in the vicinity of the thermal boundary layers that are physically sound, consistent with theory and results published by others purely from Lagrangian velocity data.

### Time series of instantaneous fields

**Fig. 5.**
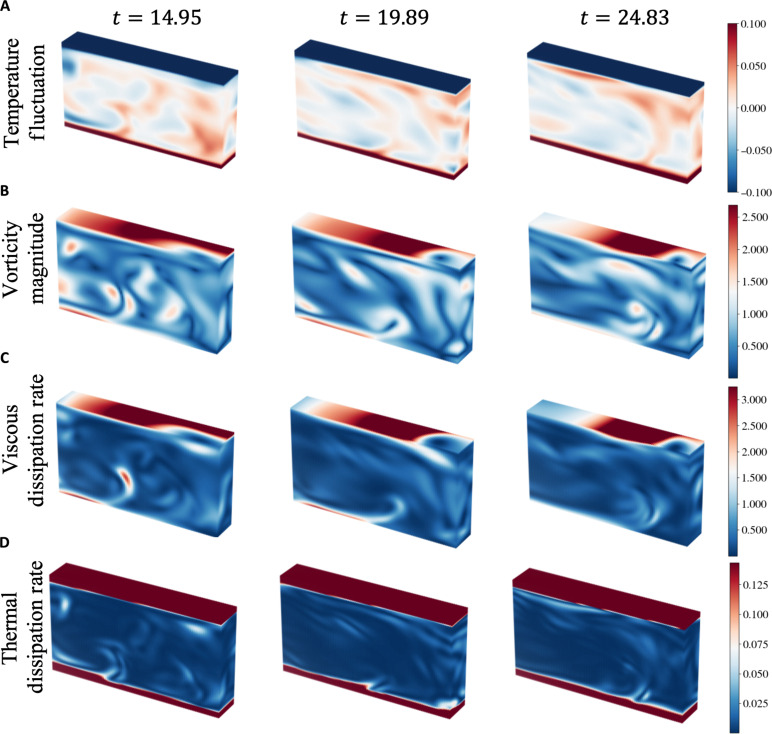
Inferred turbulent fields. Three snapshots of the inferred temperature fluctuations (**A**), vorticity magnitude (**B**), viscous dissipation rate (**C**), and thermal dissipation rate (**D**). The snapshots show the development of the flow over 10 free-fall time units. Snapshots like this are usually only available from DNS. Note the correlation between features among the different quantities. The remaining snapshots for velocity magnitude, temperature, vorticity magnitude, convective heat transfer, and thermal and viscous dissipation rate are shown in movie S2, and the corresponding temperature fluctuations and velocity and vorticity components are shown in movie S3.

Due to the continuous and differential representation of the flow by AIVT, we are not limited to reconstructing the velocity and inferring the temperature but, furthermore, are able to infer quantities requiring the gradients of these fields like vorticity, the viscous dissipation rate, and the thermal dissipation rate. To this end, we present in Fig. 5 high-resolution snapshots of the inferred temperature fluctuation Θ (A), vorticity magnitude ∣ω∣ (B), viscous dissipation rate $\varepsilon_{K}$ (C), and thermal dissipation rate $\varepsilon_{T}$ (D) for three different time instances. Note that simultaneous high-resolution snapshots of all the quantities were previously exclusive to numerical simulations. Comparing the fields of various quantities, we observe the imprint of the thermal plumes, which are associated with high kinetic energy and thermal variance. We observe high values of the vorticity magnitude and viscous dissipation rate close to the top plate. This is likely caused by the impact of the LSC, see Fig. 2 (B), resulting in strong shear and, thereby, high values of vorticity and viscous dissipation. The highest thermal dissipation occurs in the thermal boundary layers. Furthermore, the edges show that the thermal plumes are visible due to an increased thermal dissipation rate. In contrast to viscous dissipation, regions of high thermal dissipation appear more confined.

### Statistics of gradient-based quantities

**Fig. 6.**
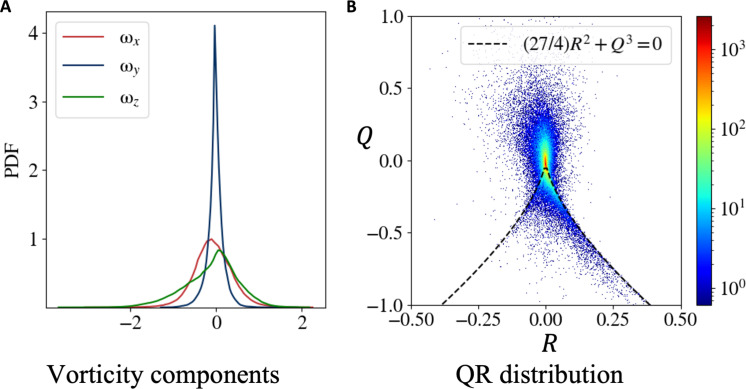
Velocity gradient-based statistics. (**A**) PDFs of the $\omega_{x}$ (red), $\omega_{y}$ (blue), and $\omega_{z}$ (green) components. $\omega_{x}$ and $\omega_{y}$ show Gaussian-like distributions, with the distribution of $\omega_{y}$ having a smaller SD; $\omega_{z}$ shows a similar width as $\omega_{x}$ but is slightly skewed. (**B**) Joint distribution of the Q and R invariants of the velocity gradient tensor. The black line indicates the Vieillefosse tail $\left(27/4\right)R^{2}+Q^{3}=0$. The joint PDF shows the typical elongated and sheared drop shape with the highest probability of low Q and R events and preference toward the right branch of the Vieillefosse tail. This shape is common for many turbulent flows, as reported by (*77*, *78*).

Because we have shown that the AIVT model provides meaningful results for gradient-related quantities of temperature and velocity, we now validate the AIVT model further by studying the statistics of these quantities. Therefore, we plot the PDFs of the $\omega_{x}$ (red), $\omega_{y}$ (blue), and $\omega_{z}$ (green) components in Fig. 6A. The PDF of $\omega_{y}$ shows a Gaussian shape and is closely distributed around 0, indicating little rotation around the vertical axis. In contrast, the PDFs of $\omega_{x}$ and $\omega_{z}$ show a wider distribution, indicating higher rotation magnitudes along the horizontal axis. This coincides with the rotation of the LSC. While the $\omega_{x}$ PDF shows almost a Gaussian shape, the negative tail of the $\omega_{z}$ PDF decreases almost linearly.

As another way to check for the consistency of the inferred gradients of the velocity field, we show the joint PDF of the Q and R invariants of the velocity tensor in Fig. 6. The dashed line indicates the Vieillefosse tail $\left(27/4\right)R^{2}+Q^{3}=0$. Q and R, which are defined in Eq. 12, represent the balance between enstrophy and strain-rate-squared magnitude and between enstrophy production and dissipation production. Thereby, the joint PDF of Q and R is a concise way to represent information about the velocity gradient (*77*). The joint PDF shows the typical elongated drop shape with the highest joint probability at Q=R=0 and a preference toward the right Vieillefosse tail. This typical shape has been observed for many turbulent flows (*77*, *78*) and proves that inferred velocity gradients are consistent with the theory.

**Fig. 7.**
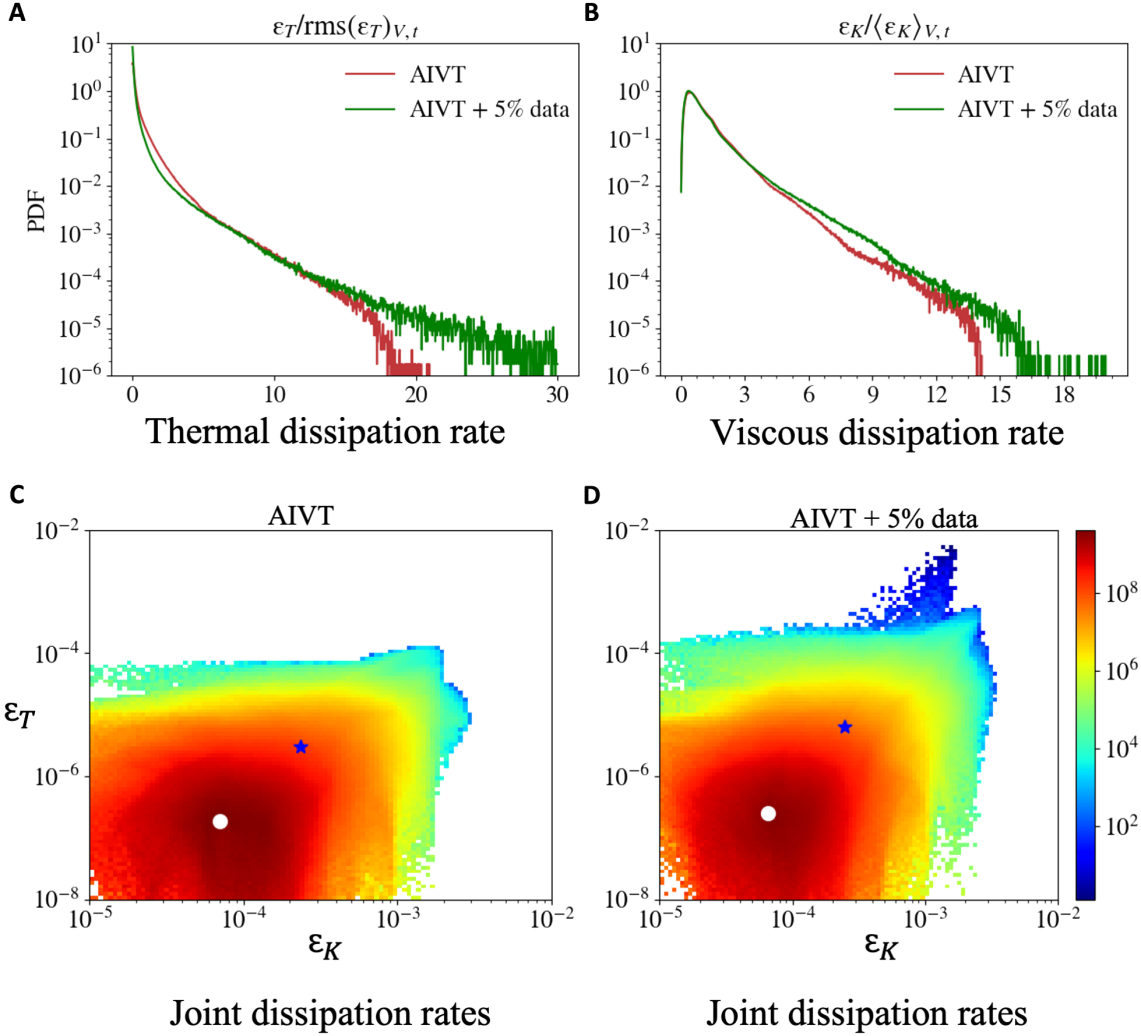
Temperature and velocity dissipation rate statistics. PDFs of the normalized thermal (**A**) and viscous (**B**) dissipation rates in the center region 0.3<y<0.7 for the AIVT model trained without (red) and with 5% temperature data (green). The PDFs of the thermal and viscous dissipation rates are similar to the results obtained from point-wise measurements of the thermal dissipation rate (*79*) and to the results obtained from local measurements of the viscous dissipation rate (*80*), respectively. Including sparse temperature observations into the training nudges the model toward higher magnitude events. Joint PDF of the thermal dissipation rates $\varepsilon_{T}$ and viscous dissipation rates $\varepsilon_{K}$ in the core region 0.3<y<0.7 of the domain obtained from the AIVT model trained only on velocity (**C**) and an additional few temperature observations (**D**). The white dot denotes the most probable joint events of $\varepsilon_{T}$ and $\varepsilon_{K}$, and the blue star denotes the intersection of ${\langle \varepsilon_{T}\rangle }_{V,t}$ and ${\langle \varepsilon_{K}\rangle }_{V,t}$, respectively. The events with the highest joint probability have lower values of $\varepsilon_{T}$ and $\varepsilon_{K}$ than the respective mean values of $\varepsilon_{T}$ and $\varepsilon_{K}$. This and the overall shape of the distribution are similar to the results obtained from DNS of RBC by Kaczorowski and Xia (*81*).

Because the AIVT model is not constrained by a finite resolution, it provides access to small-scale properties, which previously were notoriously hard to obtain from measurements, especially for LPT with limited particle image densities. Thus, we show the normalized PDF of the thermal dissipation rate ε_T_/rms(ε_T_)_V,t_ within the center region 0.3 < *y* < 0.7 inferred by the AIVT (red) and by the AIVT model trained on limited temperature data (green) in Fig. 7A. The PDF of the AIVT model purely trained on velocity data shows a higher probability for low thermal dissipation rate events compared to the PDF obtained from the AIVT model trained on limited temperature data whose tail extends further toward high-magnitude events. Again, this confirms that the AIVT model, purely trained on velocity data, tends to produce smoother results but can be notably improved by including only a few temperature observations. We compare the PDFs with normalized PDFs of point measurements of the thermal dissipation rate at *Ra* = 8.2 × 10^8^ and *Pr* = 5.4 in the center of a RBC cell reported by He *et al.* (*79*). Their results show the same trend and closely match the PDF of the AIVT model trained with a few temperature data points. This shows that our results are consistent with results obtained by other research while achieving high spatial resolution.

We proceed similarly for the viscous dissipation rate and show the normalized PDF of viscous dissipation rate ε_K_/⟨ε_K_⟩_V,t_ within the center region 0.3 < *y* < 0.7 inferred by the AIVT (red) and by AIVT model trained on limited temperature data (green) in Fig. 7B. We observe that both PDFs show the same trend and match closely up to a normalized viscous dissipation of ≈4. Beyond this point, the PDF of the AIVT model trained on velocity data only attributes a lower probability to high viscous dissipation events. This aligns with the previously observed underestimation of high-magnitude events. The comparison shows that also velocity gradient-related quantities benefit from including temperature data in the training process. To validate the results, we compare the viscous dissipation rates results of local measurements at the center of a RBC cell at *Ra* = 8.2 × 10^8^ and *Pr* = 4.34, which were recently published by Xu *et al.* (*80*). While the overall trend aligns with the literature results, the reported PDFs extend further toward high dissipation events. The difference might be, to some extent, due to the difference in *Ra* and in *Pr*.

The proposed method further allows us to investigate the joint variation of the two spatially resolved dissipation rates, a task previously only feasible with DNS. To demonstrate that, we plot the joint PDFs of the thermal and viscous dissipation rate obtained from the AIVT model in Fig. 7C and from the AIVT model trained on limited temperature data in Fig. 7D. The white dot and the blue star indicate the highest joint probability and the intersection of the individual mean of the thermal and viscous dissipation, respectively. The comparison of the joint PDFs shows a similar shape, albeit the joint PDF obtained from the AIVT model trained only on velocity tends to be offset toward lower thermal dissipation events. Additionally, the AIVT model trained with few temperature observations shows a tail of high thermal dissipation events, albeit with a very low probability. Due to the low probability of these events, they might be caused by outliers in the temperature data that propagate through into the AIVT predictions. The relative positions of the highest probability and the intersection of the individual mean dissipation rates are similar in both joint PDFs.

Because these results were so far only obtainable from DNS, we compare our results with joint PDFs of the dissipation rates obtained from DNS of RBC at $\text{Ra}=\left\{5\times 10^{6},1\times 10^{9}\right\}$ at *Pr* = 4.38 reported by Kaczorowski and Xia (*81*). Contrasting the reported joint PDF with our results, we can see that the general shape of the PDFs matches; however, they are more closely aligned to the PDF reported for *Ra* = 1 × 10^9^. While the position of the intersection of the individual mean of the dissipation rate matches, the highest joint probability is shifted by an order of magnitude toward a higher thermal dissipation rate in our results. This and the presence of events at low $\varepsilon_{K}$ and thermal dissipation rates of $\varepsilon_{T}\approx 10^{-4}$, especially in the joint PDF obtained from the model trained on temperature data, might be related to the difference in Pr.

## DISCUSSION

Here, we propose an AIVT method capable of inferring high-resolution fields and statistics of temperature, velocity, and their gradients purely from Lagrangian velocity measurements. The proposed AIVT method consists of four key components that enhance the baseline PINN model: (i) using a cKAN as a representation model instead of an MLP; (ii) reformulating the PDE into the velocity-vorticity (VV) formulation; and (iii and iv) introducing two optimization improvements, namely, RBA-R and sequential training. While PINNs have demonstrated strong performance on simulated laminar flow data (*57*), the strength of the AIVT model lies in its ability to infer hidden fields from experimental turbulent data. Table S3 presents an ablation study illustrating that our proposed modifications and extensions to previous approaches (*59*, *60*, *82*) are essential for achieving the results reported here. Additionally, we analyze the loss landscapes to demonstrate how these modifications influence the optimization process.

We trained the model using a unique set of joint Lagrangian temperature and velocity measurements, which allows us to compare the reconstructed velocity, inferred temperature, and convective heat transfer with the validated experimental data. Our results show that the reconstructed velocities are closely aligned (i.e., ~10% *RL*_2_ error) with the measured data, demonstrating the capabilities of the AIVT model to assimilate Lagrangian velocity information. Additionally, AIVT can successfully infer the temperature (i.e., ~4% *RL*_2_ error), convective heat transfer, and gradients of the temperature and velocity field purely from experimental velocity data, governing equations, and boundary conditions.

The direct comparison between the inferred and measured temperature field and statistics unveiled that AIVT captures the flow features and the increased heat transfer associated with the thermal plumes and local region of negative convective heat transfer. The inferred convective heat transfer PDF shows the typical skew toward positive values, albeit with a tendency to underestimate the event’s magnitude. Nevertheless, AIVT provides a physically consistent result of temperature and velocity, which can be further fine-tuned by including only a few temperature observations in the process.

The analysis of the vertical temperature profile showed a collapse of the measured and inferred results in the bulk region and good agreement with the theoretical results in the regions close to the plates in which the temperatures exceed the sensitivity range of the TLCs. Our study of inferred snapshots of temperature in the hot and cold boundary regions showed an agreement on the theoretical boundary layer thickness, especially in the cold boundary region, which is further verified by the vertical profile of the temperature fluctuations that matches literature results (*74*, *75*). In the hot boundary region, the imprint of detaching thermal plumes was successfully reconstructed (*72*). We demonstrated the AIVT capabilities to provide high fidelity results of various quantities by presenting snapshots of the temperature fluctuation, vorticity magnitude, and viscous and thermal dissipation rates.

Our study of the PDFs of the vorticity components unveiled enhanced vorticity magnitude along the horizontal axes, which is consistent with the orientation of the LSC. The joint distributions of the Q-R invariants of the velocity gradients tensors showed the well-known elongated drop shape common to many turbulent flows (*77*, *78*).

Our comparison of the PDFs of the thermal and viscous dissipation rates with experimental results reported by He *et al.* (*79*) and Xu *et al.* (*80*), respectively, showed qualitative agreement. Note here that their results were obtained in separate experiments at slightly different parameters by highly specialized pointwise or localized measurement. In contrast, our method provides results over the full domain height of the various quantities at arbitrary spatial-temporal resolution. Such results were previously exclusive to DNS. This is further underlined by the joint PDFs of the thermal and viscous dissipation rate, which we compared to DNS results reported by Kaczorowski and Xia (*81*). Our results show an overall similar distribution, and differences might, to some extent, be explained by differences in Rayleigh and Prandtl numbers. As for the convective heat transfer, the results can be further improved by including a few temperature observations in the training process. These can be easily achieved by standard thermocouples or resistance thermometers.

Our future research aims to improve the AIVT model further to better infer high-magnitude events. Second, we aim for a deeper integration of measurement data processing and scientific machine learning instead of treating them as different steps, thereby potentially benefiting from symbiosis effects. Last, we want to adapt our method to various other fields, measurement methods, and experimental data, e.g., magnetohydrodynamics or solid mechanics.

In summary, we proposed and applied a method that represents a paradigm shift in fluid mechanics research by fusing cutting-edge flow measurement techniques with the potential of the latest scientific machine learning methods. In combination, they can potentially change our perspective on experimental and computational methods. We think that the concept of fusing experiments and scientific machine learning is not limited to thermal convection but can be extended to fluid mechanics in general and even beyond, where the benefit of combining experimental data and scientific machine learning opens up unexplored research perspectives.

## MATERIALS AND METHODS

### Experimental temperature and velocity data collection

We obtain the joint temperature and velocity data by combining LPT and PIT. We use temperature-sensitive encapsulated TLCs to visualize RBC as described in (*24*). We performed the experiment in an equilateral hexagonal Rayleigh-Bénard convection cell of height h=60 mm and width of w=104 mm between the parallel sides. The dimension of the convection cell corresponds to an aspect ratio Γ=w/h=1.73. The sidewalls are made from glass to allow optical access, and the top and bottom plates are made from aluminum to approximate isothermal boundary conditions. We illuminated the region of interest with a white light-emitting diode and observed it by three cameras at different angles, one of which is color-sensitive, as shown in Fig. 1A. The camera setup and the illuminated domain resulted in a volume of interest of ≈80 mm (x) by 60 mm (y) by 12 mm (z) at the center of the cell. We used a water-glycerol mixture, 13% glycerol by volume, resulting in Pr=ν/κ=10.6. We applied a temperature difference of ΔT=10.3°C for the experiments, which results in $\text{Ra}=\alpha g\Delta {\mathrm{Th}}^{3}/\nu \kappa =3.4\times 10^{7}$. Here α, g, ν, and κ denote the thermal expansion coefficient, the acceleration due to gravity, height, kinematic viscosity, and thermal diffusivity, respectively.

During the experimental run, we recorded images with all cameras at a rate of 10 Hz and, afterward, processed the data as conceptualized in Fig. 1B. We obtained the particle positions and velocity from the image data by applying the Shake-the-Box algorithm (DAVIS 10.2, LaVision GmbH). Afterward, we back-projected the 3D particle positions into the image of the color camera and enforced that the back-projected and detected particle positions differ by not more than a pixel. We then extracted the 7 × 7 pixel region around the detected intensity peaks and, if necessary, centered the extracted particle images around their highest intensity peak. After recentering, we cropped the 7 × 7 regions to 5 × 5 pixel, with each region featuring an individual color particle image. We measured the particle’s temperature by using its respective color image together with the coordinate of the center pixel in the color camera image as input to our MLP, which we trained on a set of calibration images. The MLP consists of two layers with 10 neurons each and ReLU activation. To obtain the training data, we established uniform temperature distributions in the RBC cell at several reference temperature steps ranging from 19.6° to 22.6°C in 0.2°C steps. We measured each reference temperature through sensors in the plates while the color camera recorded color images. Subsequently, we extracted the individual particle images and their positions in the color camera image. We trained the neural network to predict the temperature from them, with the sensor temperature serving as a reference. We applied the trained neural network to a set of unseen test data and achieved SDs below 0.25°C for individual particles, resulting in relative uncertainties of about 8%.

We post-processed the raw measured data by applying a sliding median filter of width three that also discarded the first and last entry of every trajectory and subsequently discarded tracks shorter than five time steps, resulting in about 3000 Lagrangian joint temperature and velocity data points per snapshot. For further analysis, we non-dimensionalized the quantities by their respective characteristic units

$$\begin{array}{cc} t=\frac{t_{\text{dim}}}{\sqrt{h/\alpha \mathrm{g\Delta T}}}, & x=\left(x,y,z\right)=\frac{x_{\text{dim}}}{h},\\ T\left(x,t\right)=\frac{T_{\text{dim}}\left(x,t\right)-T_{c,\text{dim}}}{\Delta T_{\text{dim}}}, & u\left(x,t\right)=\frac{u_{\text{dim}}\left(x,t\right)}{\sqrt{h\alpha g\Delta T_{\text{dim}}}} \end{array}$$ (1)

In these equations, $t_{\text{dim}}$, $x_{\text{dim}}$_,_ $T_{\text{dim}}$, and $u_{\text{dim}}$ denote the dimension-attached time, length, temperature, and velocity. Thus, t, x T, and u refer to the time, length, temperature, and velocity in dimensionless units.

### Underlying physical laws

We consider the flow in a Rayleigh-Bénard convection cell under the Boussinesq approximation, which involves solving the full set of Navier-Stokes equations. In this study, we adopt the VV formulation, which, due to its independence from pressure, enables direct inference of temperature from sparse velocity observations and boundary conditions. As shown in fig. S6, the VP formulation induces a loss landscape with multiple local minima, complicating the optimization process. The equations of motion (eqs. S1 to S4), along with the remaining mathematical details, are provided in the Supplementary Materials.

The analyzed domain x=(x,y,z)∈Ω=(−0.6,0.6)×(0,1)×(−0.1,0.1) is a cuboid located at the center of the Rayleigh-Benard hexagonal cell (see Fig. 1A). Notice that gravity acts in the negative y direction.

#### Definition of the analyzed quantities

Here, we provide the definition of the quantities analyzed in the main part, which might not be familiar to the reader.

1) Temperature fluctuations

$$\Theta =T\left(x,t\right)-{\langle \hat{T}\rangle }_{V,t}$$ (2)

with ${\langle \hat{T}\rangle }_{V,t}$ denoting the spatial and time average of the measured temperature $\hat{T}$ over the analyzed domain of the experiment (i.e., volume and time).

2) Convective heat transfer

$$J=\sqrt{\text{Ra}\text{Pr}}\;v\left(x,t\right)\Theta \left(x,t\right)$$ (3)

3) Fluctuation profiles

$$u\prime \left(t,x\right)=u\left(t,x\right)-{\langle u\rangle }_{A,t}$$ (4)

$$v\prime \left(t,x\right)=v\left(t,x\right)-{\langle v\rangle }_{A,t}$$ (5)

$$w\prime \left(t,x\right)=w\left(t,x\right)-{\langle w\rangle }_{A,t}$$ (6)

$$T\prime \left(t,x\right)=T\left(t,x\right)-{\langle T\rangle }_{A,t}$$ (7)

where ${\langle u\rangle }_{A,t}$, ${\langle v\rangle }_{A,t}$, ${\langle w\rangle }_{A,t}$, and ${\langle T\rangle }_{A,t}$ are the mean velocities and temperature vertical profiles, which are functions of y; A is the cross-sectional area; and t is the time.

4) Thermal boundary layer thickness

$$\delta_{T}=\frac{1}{2\mathrm{Nu}}$$ (8)

with *Nu* = 22 based on *Ra* and *Pr* according to figure 7 of (*73*).

5) Viscous boundary layer thickness

$$\delta_{\nu }=\delta_{T}\sqrt{Pr}$$ (9)

6) Theoretical temperature profile for half height according to (*70*)

$$T^{*}=1-\text{exp}\left(-\delta_{T}-0.5\delta_{T}^{2}\right)$$ (10)

7) Q and R invariants of the velocity gradient tensor A

$$Q=-\frac{1}{2}A_{\mathrm{im}}A_{\mathrm{mi}}$$ (11)

$$R=-\frac{1}{3}A_{\mathrm{im}}A_{\mathrm{mn}}A_{\mathrm{ni}}$$ (12)

where $A_{\mathrm{ij}}=\frac{\partial u_{i}}{\partial x_{j}}$.

8) Viscous dissipation rate

$$\varepsilon_{K}=\frac{1}{2}\sqrt{\frac{\text{Pr}}{\text{Ra}}}{\left[\left(\nabla u\right)+{\left(\nabla u\right)}^{T}\right]}^{2}$$ (13)

9) Thermal dissipation rate

$$\varepsilon_{T}=\frac{1}{\sqrt{\text{Ra}\text{Pr}}}{\left(\text{∇}T\right)}^{2}$$ (14)

### Artificial intelligence velocimetry-thermometry

AIVT is a scientific machine learning model based on cPIKANs (*31*) and inspired by AIV (*59*, *60*) that can infer and reconstruct temperature and flow fields from experimental data and the underlying physical laws. This formulation approximates the solution of PDEs using a modified KAN (cKAN) with Chebyshev polynomials as univariate functions. In particular, a two-layer cKAN can be represented as follows

$$\text{cKAN}\left(\zeta \right)=\sum_{q=1}^{n_{1}}\Phi_{q}\left[\sum_{p=1}^{n_{0}}\phi_{q,p}\left(\zeta_{p}\right)\right]$$ (15)

where $n_{0}\;\text{and}\;n_{1}$ are the number of neurons per layer; $\zeta_{p}$ is a 1D input; and $\phi_{q,p}:\left[0,1\right]\to \mathbb{R}$, $\Phi_{q}:\mathbb{R}\to \mathbb{R}$ are learnable univariate functions.

We use AIVT to obtain continuous and differentiable flow and temperature fields from sparse velocity measurements. In particular, we approximate the solutions of the Rayleigh-Bénard equations as follows

(u,T′)=cKAN(t,x,θ) (16)

where x=(x,y,z) represents the inputs, with x,y,z as the spatial nondimensional coordinates and t as time. u=(u,v,w) is the velocity field used to derive the vorticity vector $\omega =\left(\omega_{x},\omega_{y},\omega_{z}\right)$ by using automatic differentiation (i.e., ω=∇×u). The temperature T is obtained from the predicted temperature fluctuation T′ using ADFs (*33*), which exactly satisfies the temperature boundary conditions.

We impose the remaining constraints by optimizing a combined loss function that minimizes the error from data, boundary conditions, and equations. Each loss term can be represented as

ℒC(XC,θ)=∑αmα〈[λα,rαi(xi,θ)]q〉i,where xi∈ΩC (17)

where C={D,B,E} is an index that specifies the specific loss group, namely, data ($L_{D}$), boundary ($L_{B}$) and PDE ($L_{E}$). ${\langle \cdot \rangle }_{i}$ is the mean operator of the training points $x_{i}=\left(t_{i},x_{i},y_{i},z_{i}\right)$ in the subset $X_{C}$ sampled iteratively with a probability function $p_{C,\alpha }$ from the domain $\Omega_{C}$. Here, q is a positive exponent that controls the loss function’s smoothness, enabling us to switch from $L_{2}$ to $L_{1}$ norm while training. Each loss group C controls different variables or quantities which are identified by the index α; for instance, for data loss (i.e., C=D), we constrain the velocity components so, α={u,v,w}. The details of the remaining loss terms are specified in the Supplementary Materials. The residual $r_{\alpha }\left(x_{i},\theta \right)=|\hat{\alpha }\left(x_{i}\right)-\alpha (x_{i},\theta )|$ quantifies the mismatch between the prediction $\alpha \left(x_{i},\theta \right)$ and the ideal value $\hat{\alpha }\left(x_{i}\right)$ (e.g., experimental observation) at point $x_{i}\in \Omega_{C}$. We use local weights ($\lambda_{\alpha ,i}$) to balance the point-wise contribution of the residual $r_{\alpha }\left(x_{i},\theta \right)$ and global weights ($m_{\alpha }$) to scale the averaged value of subcomponent α.

#### Sequential training

Training AIVT involves minimizing 15 objective functions, respectively, which substantially complicates the optimization process. To simplify this problem, we propose a sequential learning approach that divides the training into four stages. In the first phase, we solve a purely data-driven problem where the models learn the velocity data, boundary conditions, and an initial guess for the temperature (i.e., *T* = 0.5 in the core region 0.1<y<0.9). This initial guess is imposed weakly through a loss function, and its contribution quickly decays in the next stages, enabling the model to learn the actual temperature distribution. In the second step, we partially introduce the governing equation knowledge by optimizing a loss function that enforces a “simpler” physical law, which helps the model identify the main features of the flow. The first two stages can be considered as a guided initialization where the model learns a similar solution, facilitating and enabling convergence to the actual flow and temperature fields, which are learned during phase three. Once the model learned the desired solution, we set *q* = 1 (i.e., switch to $L_{1}$ norm), which helps refine the details of the learned flow field. The specific details of this approach can be found in the Supplementary Materials.

#### Residual-based attention with resampling

One of the main challenges in training neural networks is that the residuals (i.e., point-wise errors) may get overlooked when calculating a cumulative loss function (*43*, *64*). To address this issue, we use RBA (*41*) as local weights ($\lambda_{\alpha ,i}$), which help balance specific residuals’ point-wise contribution within each loss term α. The update rule for an RBA weight ($\lambda_{\alpha ,i}$) for the loss term α and point $x_{i}$ is based on the exponentially weighted moving average of the residuals defined as

$$\lambda_{\alpha ,i}^{\left(k+1\right)}\leftarrow {\gamma \lambda }_{\alpha ,i}^{\left(k\right)}+\eta \frac{r_{\alpha ,i}^{\left(k\right)}}{{\left\|r_{\alpha }^{\left(k\right)}\right\|}_{\infty }},i\in \left\{0,1,\ldots ,N\right\}$$ (18)

where k is the iteration, N is the number of training points, $r_{\alpha ,i}$ is the residual of loss term α for point i, η is a learning rate, and γ is a decay term that reduces the contribution of the previous iterations. This formulation induces RBA to work as an attention mask that helps the optimizer focus on capturing the spatial or temporal characteristics of the specific problem (*41*, *64*). However, RBAs are updated on the basis of the residuals calculated in a specific iteration, which may cause problems for large datasets that require batch training. This residual dependency may induce a mismatch between the network state and the generated attention mask. To address this issue, we propose using the obtained multipliers to resample the critical points. As shown in Eq. 18, RBA weights contain historical information about the high-error regions, making them suitable for defining a PDF $p_{\alpha }\left(x\right)$. Building on the previous studies (*44*, *45*), we define $p{\left(x\right)}^{\left(k\right)}$ at iteration k as follows

$$p_{\alpha }^{\left(k+1\right)}\left(x\right)=\frac{{\left(\lambda_{\alpha }^{\left(k\right)}\right)}^{\nu }}{E\left[{\left(\lambda_{\alpha }^{\left(k\right)}\right)}^{\nu }\right]}+s$$ (19)

where ${\left(\lambda_{\alpha }^{\left(k\right)}\right)}^{\nu }=\left\{\lambda_{0}^{\left(k\right)\nu },\lambda_{\alpha ,1}^{\left(k\right)\nu },\ldots ,\lambda_{\alpha ,N}^{\left(k\right)\nu }\right\}$ are the RBA weights of loss term α. The exponent ν is an integer that controls the SD of $p^{k}\left(x\right)$, and s>0 is a scalar that ensures that all points are eventually resampled. The main difference with previous approaches (*44*, *45*) is that the PDF is based on $\lambda_{i}$ instead of $r_{i}$. Because $\lambda_{i}$ is computed iteration-wise using the cumulative residuals (i.e., historical data), its corresponding PDF is more stable, which enables us to sample x at every iteration with negligible computational cost.

## Supplementary Materials

### The PDF file includes:

Supplementary Materials and Methods Supplementary Text Figs. S1 to S8 Tables S1 to S5 Legends for movies S1 to S3 References
