# Supplementary material for: AIVT: Inference of turbulent thermal convection from measured 3D velocity data by physics-informed Kolmogorov-Arnold networks
Source: Sci Adv. 2025 May 7;11(19):eads5236. doi: 10.1126/sciadv.ads5236 (PMC12057682; doi:10.1126/sciadv.ads5236)
Supplement: Supplementary file 1 — Supplementary Materials and Methods Supplementary Text Figs. S1 to S8 Tables S1 to S5 Legends for movies S1 to S3 References [file sciadv.ads5236_sm.pdf]

Science Advances
AAAS

Supplementary Materials for

AIVT: Inference of turbulent thermal convection from measured 3D velocity data by physics-informed Kolmogorov-Arnold networks

Juan Diego Toscano *et al.*

Corresponding author: Christian Cierpka, christian.cierpka@tu-ilmenau.de; George Em Karniadakis, george_karniadakis@brown.edu

Sci. Adv. **11**, eads5236 (2025)
DOI: 10.1126/sciadv.ads5236

The PDF file includes:

Supplementary Materials and Methods
Supplementary Text
Figs. S1 to S8
Tables S1 to S5
Legends for movies S1 to S3
References

Other Supplementary Material for this manuscript includes the following:

Movies S1 to S3

Materials and Methods

Underlying Physical Laws

The flow in the Rayleigh-Bénard convection cell obeys the Boussinesq approximation (i.e., Rayleigh-Bénard Equations). In this study, we use the velocity-vorticity (VV) formulation, which, given its independence of pressure, allows us to infer temperature directly from sparse velocity observations and boundary conditions. The momentum, divergence-free constraints, and energy equations are defined as follows:

$$\frac{\partial \boldsymbol{\omega}}{\partial t} + (\boldsymbol{u} \cdot \nabla)\boldsymbol{\omega} = (\boldsymbol{\omega} \cdot \nabla)\boldsymbol{u} + \sqrt{\frac{Pr}{Ra}}\nabla^2\boldsymbol{\omega} + (\nabla \times T\hat{\mathbf{e}}_g) \tag{S1}$$

$$\nabla \cdot \boldsymbol{u} = 0 \tag{S2}$$

$$\nabla \cdot \boldsymbol{\omega} = 0 \tag{S3}$$

$$\frac{\partial T}{\partial t} + (\boldsymbol{u} \cdot \nabla)T = \sqrt{\frac{1}{RaPr}}\nabla^2 T \tag{S4}$$

where $\boldsymbol{u} = (u, v, w)$ is velocity field, $\boldsymbol{\omega} = (\omega_x, \omega_y, \omega_z)$ is the vorticity and T is temperature. $\hat{\mathbf{e}}_g$ is a unit vector that defines the direction of gravity. This formulation introduces the vorticity in three dimensions, defined from Equation S5 and further constrained using the vector identity described in Equation S6.

$$\boldsymbol{\omega} = \nabla \times \boldsymbol{u} \tag{S5}$$

$$\nabla^2\boldsymbol{u} = -\nabla \times \boldsymbol{\omega} \tag{S6}$$

The analyzed domain $\boldsymbol{x} = (x, y, z) \in \Omega = (-0.6, 0.6) \times (0, 1) \times (-0.1, 0.1)$ is a cuboid located at the center of the Rayleigh-Benard hexagonal cell. And the analyzed time domain is comprised of 282 frames corresponding to 20 nondimensional times, i.e., $t \in (10, 30)$.

Kolmogorov-Arnold Networks

Kolmogorov-Arnold networks (KANs) are a type of neural network inspired by the Kolmogorov-Arnold representation theorem (*83*). This theorem states that any multivariate continuous function $f(\boldsymbol{x}) = f(x_1, x_2, \dots)$ on a bounded domain can be represented exactly as a finite composition of continuous univariate functions combined with the binary operation of addition. Motivated by this theorem, (*83*) proposed a formulation to approximate $f(\boldsymbol{x})$ as follows:

$$f(\boldsymbol{\zeta}) \approx \sum_{i_{L-1}=1}^{n_{L-1}} \Phi_{L-1,i_L,i_{L-1}} \left(\sum_{i_{L-2}=1}^{n_{L-2}} \cdots \left(\sum_{i_2=1}^{n_2} \Phi_{2,i_3,i_2} \left(\sum_{i_1=1}^{n_1} \Phi_{1,i_2,i_1} \left(\sum_{i_0=1}^{n_0} \phi_{0,i_1,i_0}(\zeta_{i_0}) \right) \right) \right) \cdots \right) \tag{S7}$$

The right-hand side of (S7) represents a KAN ($KAN(\boldsymbol{\zeta})$), where $\boldsymbol{\zeta} = (\zeta_1, \zeta_2, ...)$ is the multivariate input, L denotes the number of layers, and $\{n_j\}_{j=0}^{L}$ are the numbers of nodes (i.e., neurons) in the jth layer. The terms $\phi_{i,j,k}$ and $\Phi_{i,j,k}$ are the univariate inner and outer functions, respectively. The specific form of each $\Phi(x)$ defines variations among different KAN architectures.

Among these variations, (*31*) introduced cKANs, which utilize Chebyshev polynomials as univariate functions. In cKANs, the inner function ϕ is defined as:

$$\phi(\zeta, \theta) = \sum_n c_n T_n(\zeta),$$

where $\theta = \{c_n\}$ are trainable parameters, and $T_n : [-1, -1] \rightarrow \mathbb{R}$ is the nth-order Chebyshev polynomial, recursively defined as

$$T_n(\zeta) = 2\zeta T_{n-1}(\zeta) - T_{n-2}(\zeta).$$

This recursive formulation ensures numerical stability by generating higher-order terms systematically from lower-order polynomials. Similarly, the outer functions are defined as:

$$\Phi(\zeta, \theta) = \phi(\tanh(\zeta)).$$

As described in (*31*), this normalization ensures that the inputs to each univariate function remain within the range $[-1, 1]$, which is necessary since Chebyshev polynomials are defined over this interval. This constraint improves numerical stability and preserves the accuracy of polynomial expansions. Previous studies have shown that cKANs are robust to noise (*31*) and accelerated convergence for challenging PDES such as the Allen Cahn equation (*61*).

cKANs and MLPs relation While the Kolmogorov-Arnold Representation Theorem (KART) describes a decomposition of multivariate functions into sums of univariate functions which can be non-smooth or even fractal (*84*), in practice some Kolmogorov-Arnold Networks (KANs) (*83*) formulations deviate from this framework by employing nested formulations that prioritize approximation over exact representation (*31, 61*). A less disruptive interpretation was proposed in (*85*),

where KANs are described as multi-head MLPs. Interestingly, one of the initial theoretical analyses of KART's approximation capabilities (*86*) employed combinations of sigmoid functions, resulting in a formulation closely resembling a single hidden layer MLP.

Following this framework, we demonstrate that a two-layer Chebyshev KAN (cKAN) can be interpreted as a single hidden layer MLP with Chebyshev feature expansions applied to both the input and output layers. This extension appears natural, as input feature expansions have been shown to mitigate spectral bias in MLPs (*32*) and improve accuracy (*87–92*). Consequently, extending this idea to output expansions is a logical step, potentially enhancing performance from the adaptive-basis perspective of MLPs (*93*). In this framework, an output Chebyshev expansion introduces structured sparsity and orthogonality, which may further improve model efficiency.

Definition: Define the *one-dimensional Chebyshev feature expansion* as a map $E : [-1, 1] \to \mathbb{R}^{k+1}$ that expands a univariate input as:

$$E(x_p) = \begin{bmatrix} T_0(x_p) \\ T_1(x_p) \\ \vdots \\ T_k(x_p) \end{bmatrix} \in \mathbb{R}^{k+1},$$

where each entry $T_i(x)$ is the corresponding Chevysehv polynomial of order i where k is the maximum polynomial degree.

Definition: Define the *Chebyshev feature expansion* as an extension map $F : [-1, 1]^d \to \mathbb{R}^{d(k+1)}$ that encodes a multidimensional input into a higher dimensional space that is:

$$F(\mathbf{x}) = \text{concat}(E(x_1), \ldots, E(x_d)) \in \mathbb{R}^{d(k+1)}.$$

where $E(x_i)$ is the corresponding one-dimensional Chebyshex expansion of variable x_i.

Claim 1: Let $\mathbf{x} = \{x_1, \ldots, x_d\} \in [-1, 1]^d$ be a d−dimensional input and $\boldsymbol{y}$ be an m-dimensional output of a 2-layer Chebyshev KAN, $cKAN : [-1, 1] \to \mathbb{R}^m$ defined as:

$$y_j = cKAN(\mathbf{x})_j = \sum_{q=1}^{n+1} \Phi_{q,j}\left(\sum_{p=1}^{d} \phi_{q,p}(x_p)\right). \tag{S8}$$

where y_j denotes the $j-$th entry of $\boldsymbol{y} \in \mathbb{R}^m$. Now, there exist weight matrices $W^{(1)}$ and $W^{(2)}$ such that a 2-layer $cKAN$ can be expressed as one-hidden layer MLP whose input and output have been expanded using a Chebyshev feature expansion $F : [-1, 1]^d \longrightarrow \mathbb{R}^{d(k+1)}$, that is $eMLP : [-1, 1] \rightarrow \mathbb{R}^m$:

$$\boldsymbol{y} = eMLP(\boldsymbol{x})$$
$$\boldsymbol{y} = W^{(2)} \cdot F\sigma(W^{(1)} \cdot F(\boldsymbol{x}))$$

with activation function $\sigma = \tanh$.

Proof of Claim: Consider a two-layer cKAN with d inputs, where $\mathbf{x} = \{x_1, \ldots, x_d\} \in [-1, 1]^d$, and a m-dimensional output $\boldsymbol{y}$, thus, the j entry of the y would given by equation S8. By definition (*31*), the *inner function* $\phi_{q,p}$ is a univariate function of x_p, expressed as a Chebyshev expansion:

$$\phi_{q,p}(x_p) = \sum_{i=1}^{k+1} C^{(1)}_{q,p,i} T_{i-1}(x_p),$$

where $T_{i-1} : [-1, 1] \longrightarrow \mathbb{R}$ denotes a Chebyshev polynomial of degree $i - 1$. Substituting this expansion into (S8), we obtain:

$$y_j = \sum_{q=1}^{n+1} \Phi_{q,j} \left(\sum_{p=1}^{d} \sum_{i=1}^{k+1} C^{(1)}_{q,p,i} T_{i-1}(x_p) \right).$$

This formulation implies that the transformation consists of (1) expanding the input via $T_{i-1}(x_p)$, (2) scaling each term by $C^{(1)}_{q,p,i} T_{i-1}(x_p)$, (3) summing across the expansion terms, and (4) summing over the input dimensions. Due to the associative property of summation, the last two steps can be combined into a single sum easily described as matrix-vector multiplication.

Towards this end, extend the inputs using a Chebyshev feature expansion:

$$F(\mathbf{x}) = \mathrm{concat}(E(x_1), \ldots, E(x_d)) \in \mathbb{R}^{d(k+1)}.$$

Then, let $t = (p-1)(k+1)+i$ and rewrite the inner function in vectorized form as:

$$\sum_{p=1}^{d} \phi_{q,p}(x_p) = \sum_{p=1}^{d}\sum_{i=1}^{k+1} W^{(1)}_{q,(p-1)(k+1)+i} F(\mathbf{x})_{(p-1)(k+1)+i}$$
$$\sum_{p=1}^{d} \phi_{q,p}(x_p) = \sum_{t=1}^{d(k+1)} W^{(1)}_{q,t} F(\mathbf{x})_t$$
$$\sum_{p=1}^{d} \phi_{q,p}(x_p) = \left(W^{(1)} \cdot F(\mathbf{x})\right)_q$$

where $W^{(1)} \in \mathbb{R}^{(n+1)\times d(k+1)}$ is a matrix encoding the coefficients $C^{(1)}_{q,p,i}$, namely $W^{(1)}_{q,(p-1)(k+1)+i} = C^{(1)}_{q,p,i}$. Substituting this into (S8), we rewrite the model as:

$$y_j = \sum_{q=1}^{n+1} \Phi_{q,j}\left(W^{(1)} \cdot F(\mathbf{x})\right)_q.$$

As described in the original study on cKANs (*31*), to ensure numerical stability in the Chebyshev expansion (i.e., $T_{i-1} : [-1,1] \to \mathbb{R}$), the *outer functions* Φ_q are defined as a composition with a hyperbolic tangent that is $\Phi_q(\cdot) = \phi_q(\tanh(\cdot))$ which leads to:

$$y_j = \sum_{q=1}^{n+1} \phi_{q,j}\left(\tanh\left(W^{(1)} \cdot F(\mathbf{x})\right)\right)_q. \tag{S9}$$

Under this transformation, the sum of inner functions,

$$\sum_{p=1}^{d} \phi_{q,p}(x_p) = \tanh(W^{(1)} \cdot F(\mathbf{x}))_q,$$

resembles a set of *perceptrons*, the fundamental building blocks of an MLP, as it consists of a linear layer followed by an activation function. In particular, if the outer function were simply a linear layer $\phi_{q,j}(\cdot) = W^{(2)}_{q,j}\cdot$, the model would recover an MLP formulation without bias:

$$y_j = \sum_{q=1}^{n+1} W^{(2)}_{q,j}\left(\tanh(W^{(1)} \cdot F(\mathbf{x}))\right)_q,$$
$$y_j = \left(W^{(2)} \cdot \tanh(W^{(1)} \cdot F(\mathbf{x}))\right)_j.$$

For the general case, we interpret cKANs as an MLP with both input and output Chebyshev expansions. Denoting the transformed inner sum as:

$$\xi_q = \tanh(W^{(1)} \cdot F(\mathbf{x}))_q,$$

and expanding the *outer function* $\phi_q(\cdot)$ in the Chebyshev basis:

$$\phi_{q,j}(\xi_q) = \sum_{i=1}^{d+1} C^{(2)}_{q,i,j} T_{i-1}(\xi_q),$$

we rewrite the output function as:

$$y_j = \sum_{q=1}^{n+1} \sum_{i=1}^{d+1} C^{(2)}_{q,i,j} T_{i-1}(\xi_q).$$

Once again, due to the associative property of summation, we perform it concurrently by defining:

$$F(\boldsymbol{\xi}) = \text{concat}(E(\xi_1), \ldots, E(\xi_{n+1})) \in \mathbb{R}^{m\times(n+1)(k+1)},$$

Now denote $s = (q-1)(k+1) + i$ and rewrite the output in matrix form:

$$y_j = \sum_{q=1}^{n+1} \sum_{i=1}^{k+1} W^{(2)}_{(q-1)(k+1)+i,j} F(\boldsymbol{\xi})_{(q-1)(k+1)+i} \tag{S10}$$

$$y_j = \sum_{s=1}^{(n+1)(k+1)} W^{(2)}_{s,j} F(\boldsymbol{\xi})_s \tag{S11}$$

$$y_j = W^{(2)} \cdot F(\boldsymbol{\xi}) \tag{S12}$$

where $W^{(2)}$ encodes the outer Chebyshev coefficients $W^{(2)}_{(q-1)(k+1)+i,j} = C^{(2)}_{q,i,j}$.

Thus, the final formulation of cKAN is given by:

$$\boldsymbol{y} = W^{(2)} \cdot F\Big(\tanh(W^{(1)} \cdot F(\mathbf{x}))\Big)$$

Notice that the RHS is identical to an MLP with input and output Chebyshev feature expansions when $\sigma = \tanh$:

$$\boldsymbol{y} = W^{(2)} \cdot F\sigma(W^{(1)} \cdot F(\mathbf{x}))$$
$$\boldsymbol{y} = eMLP(\mathbf{x})$$

■

We then extend these results to show that a $2L$-layer nested cKAN can be regarded as an L-layer nested MLP in which each input, intermediate, and output layer undergoes Chebyshev expansions. This extension is also intuitive, as similar techniques, such as adaptive activation functions (*94*), have successfully incorporated frequency- and scale-dependent transformations to enhance model expressivity (*94, 95*).

Claim 2: A nested $2L$ layer nested cKAN:

$$\boldsymbol{y} = \Phi^{(2L)} \circ \Phi^{(2L-1)} \circ \cdots \Phi^{(1)} \circ \phi(\boldsymbol{x})$$

can be regarded as a L-layer nested MLP in which each input, intermediate, and output layer are further expanded using Chebyshev expansions:

$$\boldsymbol{y} = eMLP^{(L)} \circ eMLP^{(L-1)} \circ \cdots \circ eMLP^{(1)}(\boldsymbol{x})$$

Proof of claim: Follows from Claim 1 noticing for any layer $k \in \{0, 1, \cdots, L\}$ with inputs $\boldsymbol{z} \in \mathbb{R}^n$ for some widht $n \in \mathbb{N}$:

$$eMLP^{(k)}(\boldsymbol{z}) = \Phi^{(2k+1)} \circ \Phi^{(2k)}(\boldsymbol{z})$$

■

Notice that Claim 2 provides an intuitive explanation for why cKANs may require fewer parameters. The Chebyshev expansion introduces structured sparsity and orthogonality, which could enhance feature expressivity and reduce redundancy in learned representations. However, formal proof of this property remains an open question and is beyond the scope of the present study.

Artificial Intelligence Velocimetry Thermometry (AIVT)

AIVT is a scientific machine learning model based on cPIKANs (*31*) and inspired on AIV (*59, 60*) that can infer and reconstruct flow fields from experimental data and the underlying physical laws. We use AIVT to obtain continuous and differentiable flow and temperature fields from sparse velocity measurements. In particular, we approximate the solutions of the Rayleigh-Bénard equations as follows:

$$(\boldsymbol{u}, T') = cKAN(t, \mathbf{x}, \theta) \tag{S13}$$

where $\mathbf{x} = (x, y, z)$ represents the inputs namely, x, y, z as the spatial nondimensional coordinates, t as time. $\boldsymbol{u} = (u, v, w)$ is the velocity field used to derive the vorticity vector $\boldsymbol{\omega} = (\omega_x, \omega_y, \omega_z)$ by applying the curl operator (i.e., $\boldsymbol{\omega} = \nabla \times \boldsymbol{u}$). This reformulation strictly satisfies Equation S3, as the divergence of the curl of any vector field equals zero. The temperature T is obtained from the predicted temperature fluctuation T' as follows:

$$T(t, \mathbf{x}, \theta) = g(\mathbf{x}) + \varphi(\mathbf{x})T'(t, \mathbf{x}, \theta) \tag{S14}$$

Here, $g(\mathbf{x})$ is a function that satisfies the boundary conditions, and $\varphi(\mathbf{x})$ is a distance function that equals zero at the boundaries. In this study, we define $g(\mathbf{x})$ and $\varphi(\mathbf{x})$ as follows:

$$g(\mathbf{x}) = g(y) = T_h - y\frac{(T_h - T_c)}{H} \tag{S15}$$

$$\varphi(\mathbf{x}) = \varphi(y) = (y - H)(y) \tag{S16}$$

where $T_h = 1$ and $T_c = 0$ are the hot and cold plate's temperature, and $H = 1$ is the eight of the Rayleigh Benard cell. As described in (*33*), this formulation allows us to exactly enforce the temperature boundary conditions, with $T|_{y=1} = 1$ and $T|_{y=0} = 0$.

We impose the remaining constraints by optimizing a combined loss function that minimizes the error from data, boundary conditions, and equations. The data loss ($\mathcal{L}_D$) controls the mismatch between the network prediction and experimental observations and is explicitly defined as:

$$\mathcal{L}_D(X_D, \theta) = \sum_d m_d \langle [\lambda_{d,i} r_d(\mathbf{x}_i, \theta)]^q \rangle_i, \text{ where } \mathbf{x}_i \in \Omega_D \tag{S17}$$

where $\langle \cdot \rangle_i$ denotes the mean operator, q is a positive exponent that controls the smoothness of the loss, and $\mathbf{x}_i = (t_i, x_i, y_i, z_i)$ are the data points from the subset X_D sampled from the data domain

Ω_D. The index $d = \{u, v, w\}$ identifies the specific variables constrained in the loss, where u, v, w represent the velocities in the x, y, z directions. The residual $r_d(\mathbf{x}_i, \theta) = |\hat{d}(\mathbf{x}_i) - d(\mathbf{x}_i, \theta)|$ quantifies the difference between the experimental observation $\hat{d}(\mathbf{x}_i)$ and the network prediction $d(\mathbf{x}_i, \theta)$ at point $\mathbf{x}_i \in \Omega_D$. We use local weights ($\lambda_{d,i}$) to balance the point-wise contribution of the residual $r_d(\mathbf{x}_i, \theta)$ and global weights (m_d) to scale the averaged value of subcomponent d.

The velocity and vorticity boundary conditions are imposed by the boundary loss ($\mathcal{L}_B$), described in Equation S18.

$$\mathcal{L}_B(X_B, \theta) = \sum_b m_b \langle [\lambda_{b,j} r_b(x_j, \theta)]^q \rangle_j, \text{ where } x_j \in \Omega_B \tag{S18}$$

here, $\Omega_B = \{(t, x, y, z) \in \Omega; y = 0 \text{ or } y = 1\}$ is the boundary domain corresponding to the top and bottom walls of the domain Ω. To impose the no-slip boundary conditions and no-normal vorticity, we set $b = \{u, v, w, \omega_y\}$, with residuals $r_b(x_j, \theta) = |b(x_j, \theta)|$, local weights $\lambda_{b,j}$, and global weights m_b.

To enforce our governing equations, we rewrite equations S1, S3, S4 and S6 in their residual form and impose them iteratively by minimizing the loss function described in equation S24. In the following section, we follow (*38*) and use subscript notation to define the derivative with respect to each component (e.g., $u_z = \frac{\partial u}{\partial z}$). The residuals for the momentum and temperature residual equations are defined as:

$$r_{Mx} = a_t + ua_x + va_y + wa_z - \left(au_x + bu_y + cu_z + \left(\frac{Pr}{Ra}\right)^{1/2} (a_{xx} + a_{yy} + a_{zz}) - T_z\right) \tag{S19}$$

$$r_{My} = b_t + ub_x + vb_y + wb_z - \left(av_x + bv_y + cv_z + \left(\frac{Pr}{Ra}\right)^{1/2} (b_{xx} + b_{yy} + b_{zz})\right) \tag{S20}$$

$$r_{Mz} = c_t + uc_x + vc_y + wc_z - \left(aw_x + bw_y + cw_z + \left(\frac{Pr}{Ra}\right)^{1/2} (c_{xx} + c_{yy} + c_{zz}) + T_x\right) \tag{S21}$$

$$r_T = T_t + uT_x + vT_y + wT_z - \left(\frac{1}{PrRa}\right)^{1/2} (T_{xx} + T_{yy} + T_{zz}) \tag{S22}$$

$$\tag{S23}$$

where $a = w_y - v_z$, $b = u_z - w_x$ and $c = v_x - u_y$ are the vorticity components in the x, y and z directions. Similarly, we rewrite the conservation of mass and the vector identity (equation S6) into their three components as follows:

$$r_{DFu} = u_x + v_y + w_z$$
$$r_{VIx} = c_y - b_z + (u_{xx} + u_{yy} + u_{zz})$$
$$r_{VIy} = a_z - c_x + (v_{xx} + v_{yy} + v_{zz})$$
$$r_{VIz} = b_x - a_y + (w_{xx} + w_{yy} + w_{zz})$$

We enforce the physical knowledge by minimizing the residual equations using a combined loss function:

$$\mathcal{L}_E(X_E, \theta) = \sum_e m_e \langle [\lambda_{e,l} r_e(x_l, \theta)]^q \rangle_l, \text{ where } x_l \in \Omega \tag{S24}$$

here, $e = \{M_x, M_y, M_z, DF_u, T, VI_x, VI_y, VI_z, \}$ identifies the residuals from the momentum M_β, continuity DF, temperature T, and the vector identities VI in the $\beta = \{x, y, z\}$ directions. We define the residual for subcomponent e and point $x_l \in \Omega$ as $r_e(x_l, \theta) = |e(x_l, \theta)|$, and balance its point-wise and averaged contribution to $\mathcal{L}_E$ using local multipliers $\lambda_{e,l}$ and global weights m_e, respectively. The remaining constraint (i.e., equation S3) is strictly satisfied by the model's definition.

Sequential Training

Training AIVT involves minimizing 15 objective functions, respectively, which significantly complicates the optimization process. Notably training this problem directly using a baseline model (*59, 60, 82*) would lead to a suboptimal performance as shown in figure S1(A). To simplify this problem, we propose a sequential learning approach that divides the training into four stages (See figure S1(B)). To ensure a smooth transition, we reinitialize the optimizer and local multipliers at the beginning of each phase.

In the first stage, we solve a purely data-driven problem where the models only learn the data and the boundary conditions. In particular, for the first (1/6) of the training iterations, we minimize the following loss function:

$$\mathcal{L} = \mathcal{L}_D + \mathcal{L}_B + \mathcal{L}_T^* \tag{S25}$$

Where the data loss $\mathcal{L}_D$ and the boundary loss $\mathcal{L}_B$ are described in equations S17 and S18 with $q = 2$ (i.e., Mean Squared error). $\mathcal{L}_T^*$ is a constraint that guides the model during the initial iterations. In particular, this loss guides the predicted temperature in the core region $(0.1 < y < 0.9)$ to match the theoretical average $\bar{T} = 0.5$. This constraint is imposed softly since it's scaled with a small global weight $m_T^* = m_d/100$ that decays as we approach the final iterations. Our results have shown that using $\mathcal{L}_T^*$ is not necessary to reproduce our results; however, it helps stabilize the model for different initialization.

In the second step $(1/6 - 2/6)$, we partially include the equation constraints as:

$$\mathcal{L} = \mathcal{L}_D + \mathcal{L}_B + \mathcal{L}_T^* + \mathcal{L}_E^* \tag{S26}$$

$\mathcal{L}_E^*$ is a loss function that enforces "partial physics." $\mathcal{L}_E^*$ shares equation S24 formulation but differs from it since it enforces the PDE equations using a lower Rayleigh number $Ra = Ra/100$. This technique enables us to learn and capture the diffusive features of the flow. The first two stages can be considered an initialization or "warm-up" stage, where the model learns a similar function, facilitating and enabling convergence to the actual solution. The global weights used during these pre-training phases are described in Table S4.

In the third stage, $(2/6 - 5/6)$ of the training iterations, we use the final Rayleigh number and learn the turbulent flow by minimizing the full loss function:

$$\mathcal{L} = \mathcal{L}_D + \mathcal{L}_B + \mathcal{L}_E \tag{S27}$$

where $\mathcal{L}_E$ is described in equation S24. In the last stage (i.e., last quarter), we set $q = 1$, which switches the MSE to the mean absolute error MAE, helping refine the details of the learned solution. The global weights used in these training stages are detailed in Table S5.

Residual-Based Attention with resampling (RBA-R)

One of the main challenges in training neural networks is that the residuals (i.e., point-wise errors) may get overlooked when calculating a cumulative loss function (*41, 43*). To address this issue, research has proposed using direct and indirect methods. Direct methods employ local weights($\lambda_{\alpha,i}$) to balance specific residuals' point-wise contribution within each loss term α. On the other hand, indirect methods rely on resampling or refining the high-error regions (*45*). In this study, we extend

Table S1: **Detailed results.** Relative L_2 error (RL_2) on the validation dataset.

Method	RL_2 u(%)	RL_2 v (%)	RL_2 w(%)	RL_2 T (%)
AIVT	9.68 ± 0.23	10.86 ± 0.073	12.00 ± 0.13	3.62 ± 0.89
AIVT+5%Data	9.94	10.01	12.32	2.76

residual-based attention (RBA) weights to scale the point-wise residual (i.e., direct method) and to resample the high error regions (i.e., direct method). The update rule for an RBA weight ($\lambda_{\alpha,i}$) for the loss term α and point x_i is based on the exponentially weighted moving average of the residuals defined as:

$$\lambda_{\alpha,i}^{(k+1)} \leftarrow \gamma\lambda_{\alpha,i}^{(k)} + \eta\frac{r_{\alpha,i}^{(k)}}{\|\boldsymbol{r}_{\alpha}^{(k)}\|_{\infty}}, \quad i \in \{0, 1, ..., N\}, \tag{S28}$$

where k is the iteration, N is the number of training points, $r_{\alpha,i}$, is the residual of loss term α for point i, η is a learning rate, and γ is a decay term that reduces the contribution of the previous iterations. This formulation induces RBA to work as an attention mask that helps the optimizer focus on capturing the spatial or temporal characteristics of the specific problem (*41, 64*).

Generally, obtaining an attention mask requires processing as many multipliers as training points, which may hinder its optimal application for large datasets due to its computational cost. To address this issue, we propose using the obtained multipliers to resample the critical points. As shown in equation S28, RBA weights contain historical information about the high-error regions, making them suitable for defining a probability density function $p_\alpha(\mathbf{x})$. Building on the previous studies (*44, 45*), we define $p(\mathbf{x})^{(k)}$ at iteration k as follows:

$$p_{\alpha}^{(k+1)}(\mathbf{x}) = \frac{(\boldsymbol{\lambda}_{\alpha}^{(k)})^{\nu}}{\mathbb{E}[(\boldsymbol{\lambda}_{\alpha}^{(k)})^{\nu}]} + s \tag{S29}$$

where $(\boldsymbol{\lambda}_{\alpha}^{(k)})^{\nu} = \{\lambda_{0}^{(k)\nu}, \lambda_{\alpha,1}^{(k)\nu}, ..., \lambda_{\alpha,N}^{(k)\nu}\}$ are the RBA weights of loss term α. The exponent ν is an integer that controls the standard deviation of $p^k(\mathbf{x})$, and $s > 0$ is a scalar that ensures that all points are eventually resampled.

Supplementary Text

Reconstructed Velocity

We validate our model performance on the core region ($0.1 < y < 0.9$) of the validation data (i.e., 50% of the velocity measurements). The AIVT's relative L_2 error

$$RL_2 = \frac{\|\hat{d}(x) - d(x)\|_2}{\|\hat{d}(x)\|_2} = \frac{\sqrt{\sum_{i=1}^{n} \left(\hat{d}(\mathbf{x}_i) - d(\mathbf{x}_i, \theta)\right)^2}}{\sqrt{\sum_{i=1}^{n} \hat{d}(\mathbf{x}_i)^2}} \tag{S30}$$

in the core region ($0.1 < y < 0.9$) for five different seeds are presented in Table S1. Notice that the uncertainty (i.e., three standard deviations) from the model parameters is less than 0.25%. Figure S1(C) shows the best-performing model RL_2 and L_∞ i.e., maximum absolute difference

$$L_\infty = \|\hat{d}(\mathbf{x}_i) - d(\mathbf{x}_i, \theta)\|_\infty = \max(|\hat{d}(\mathbf{x}_i) - d(\mathbf{x}_i, \theta)|), \tag{S31}$$

indicating that the error value is consistent for the analyzed time steps (i.e., 282 frames). The pointwise error distribution for the worst case is shown in figure S7, and the results for the remaining time steps are shown in Movie S1.

Inferred Temperature

Using AIVT, we reconstruct the temperature field purely from the sparse velocity information. To validate these results, we use all the temperature information collected using the proposed experimental method. The AIVT mean RL_2 error is 3.62%, and as shown in Table S1, the uncertainty due to the model parameters is less than 1%.

To improve our framework performance, we introduce a second network that uses 5% of the velocity and temperature for training and validated with the remaining unseen data. As shown in table S1, even with 5% data, we can reduce the temperature relative L_2 error to 2.76%. Also, notice that reducing the velocity training data has a small influence on the velocity reconstructions.

To further investigate our results, we compute the normalized root mean squared (σ/σ_{max}) velocity and temperature fluctuations (See figure S2 along the whole domain. The vertical dashed black lines indicate the thermal boundary layer thickness, while the solid vertical black lines indicate the viscous boundary layer thickness.

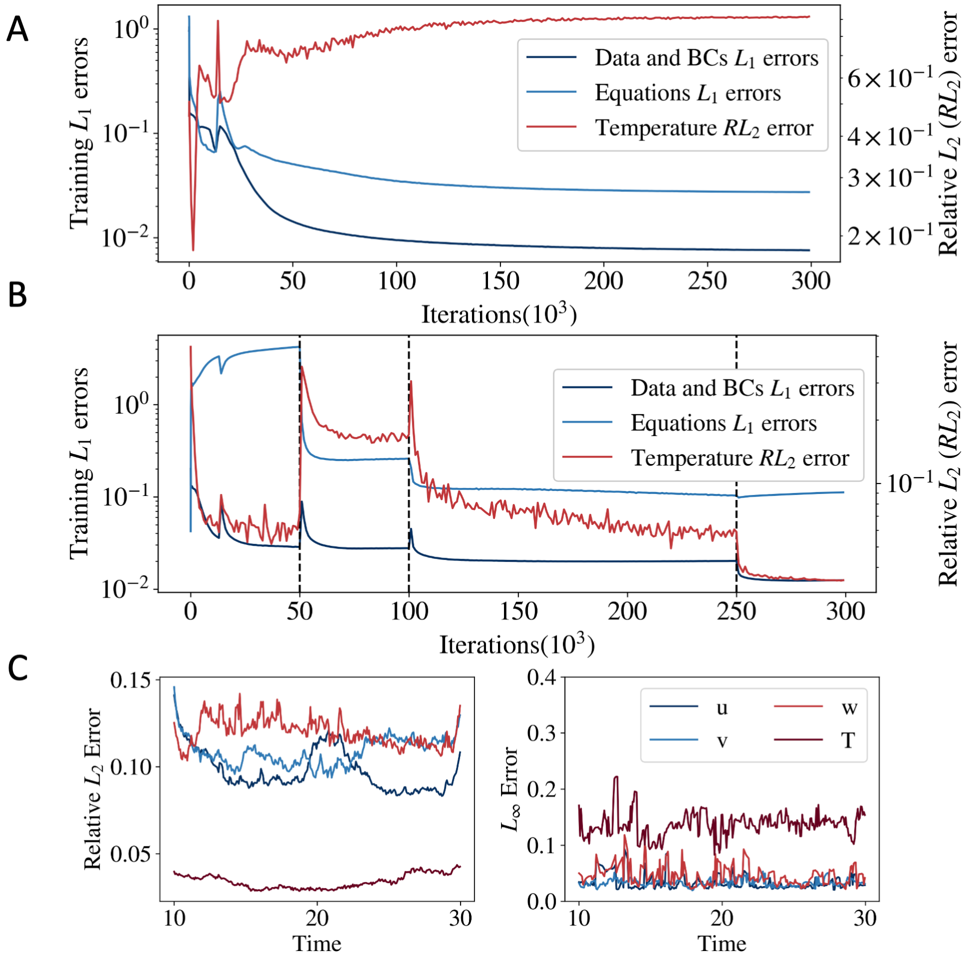

Figure S1: **AIVT Performance Comparison**. (A) Baseline model performance: The temperature relative L_2 error on the validation dataset is shown in red (right axis), while the training L_1 errors are displayed in blue (left axis). Despite a decrease in training error, the validation error remains high, indicating poor generalization. (B) Sequential training approach: The problem is initially reduced to a data-driven phase, where the model learns velocity information, boundary conditions, and the theoretical temperature profile. While the temperature relative L_2 error (in red) is low at this stage, equation errors remain high. In the second step, the model incorporates physics by training on the governing equations at a lower Rayleigh number (Ra). In the third stage, the full model is trained. Finally, in the last stage, solutions are refined by adjusting the loss exponent to $q = 1$, imposing an L_1 norm, which enhances accuracy. (C) Relative L_2 and L_∞ (maximum absolute difference) errors for the analyzed timesteps in the proposed framework.

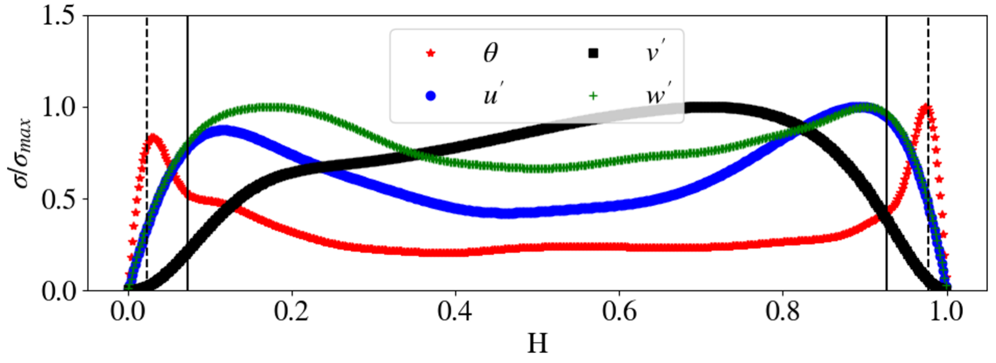

Figure S2: **Fluctuations** Normalized root-mean-squared ($\sigma/(\sigma)_{\max}$) velocity u', v', w' temperature T' fluctuations on the whole domain. The vertical dashed lines indicate the viscous δ_ν and thermal δ_T boundary layer thicknesses, respectively.

AIVT outputs continuous and differentiable functions that enable us to define the velocity, temperature, or derived fields (i.e., convective heat transfer, thermal and viscous dissipation rates) at any point inside the domain. The predictions of these quantities in a dense Eulerian grid are shown in Figure S8 for a representative time, and the results for all time steps are shown in Movies S2 and S3.

MLPs and cKANs Comparison

To allow a fair comparison between cKAN and MLP-based formulations, we choose an architecture that approximately matches the total number of parameters $|\theta|$, which can be quantified as follows (*31*):

$$|\theta|_{MLP} = L_{MLP}\left[I + (n_l - 1)L_{MLP} + O\right] \tag{S32}$$

$$|\theta|_{MLP} \sim O(n_l L_{MLP}^2), \tag{S33}$$

$$|\theta|_{cKAN} = L_{cKAN}\left[I(k+1) + (n_l - 1)L_{cKAN}(k+1) + O(k+1)\right] \tag{S34}$$

$$|\theta|_{cKAN} \sim O(n_l L_{cKAN}^2(k+1)). \tag{S35}$$

Here, I and O represent the number of inputs and outputs, respectively, while n_l denotes the number of hidden layers, L is the number of neurons per hidden layer, and k is the polynomial

order, chosen as $k = 5$ based on previous studies (*31*). Therefore, fixing L_{MLP}, the number of parameters in cKANs can be approximately matched by setting $L_{cKAN} = L_{MLP}/\sqrt{k+1}$. Similarly, fixing L_{cKAN}, the equivalent number of parameters for an MLP would be $L_{MLP} = L_{cKAN}\sqrt{k+1}$.

To enable a fair comparison of the representation capabilities of both models, we evaluate the performance of MLP and cKAN architectures across different numbers of layers. Initially, we consider $n_l = \{3, 5, 7\}$ and set $L_{cKAN} = 64$ and $L_{MLP} = 157$, resulting in parameter counts of approximately $|\theta| \approx \{50k, 100k, 150k\}$. Additionally, since MLPs benefit from higher parameter counts, we include an additional case using the MLP architecture employed in previous AIV studies (*59, 60, 82*), where MLP is defined with $n_l = 8$ and $L_{MLP} = 200$, which corresponds to $L_{cKAN} = 81$ for an equivalent comparison. Following prior studies, we use $\tanh(\cdot)$ as the activation function, with an exponentially decaying learning rate, starting at $lr_0^{(MLP)} = 10^{-3}$ and decreasing to $lr_f^{(MLP)} = 10^{-5}$ with a decay rate of 0.9. As noted in (*31*), cKANs generally benefit from lower learning rates, so we set $lr_0^{(cKAN)} = lr_0^{(MLP)}/3 \approx 0.3 \times 10^{-3}$.

All models in this comparison were trained using the ADAMW optimizer (*96*) and incorporated the full set of enhancements proposed in this paper, including the velocity-vorticity formulation, sequential training, and Residual-Based Attention with Resampling (RBA-R) with $\gamma = 0.999$, $\eta = 0.1$, $\nu = 2.0$, and $s = 0.5$ (see Equation 19 in the main text). Training was conducted on a single NVIDIA A100-SMXM4-80GB GPU, ensuring a fair and consistent comparison between cKAN and MLP architectures across different network configurations.

Training Data Our experimental data is obtained as described in the preceding sections. From this dataset, we use 50% of the sparse velocity data for training, corresponding to $N_D = 381{,}955$ points. To effectively enforce the top and bottom boundary conditions, we utilize $N_B = 100{,}000$ points. Finally, to impose the PDE constraints, we employ $N_c = 820{,}000$ collocation points, sampled using Latin hypercube sampling, as commonly done in previous PIML studies (*30*).

For DNS of thermal convection, the resolution is typically set to approximately the Kolmogorov scale for $Pr < 1$ or the Batchelor scale for $Pr > 1$ (*97*). We estimate the dimensionless mean Kolmogorov scale and mean Batchelor scale of the experiment to be approximately 0.02 and 0.006 times the domain height, respectively. Resolving the mean Batchelor scale within the measured domain would require around 1,000,000 grid points, which is comparable to the number of collocation

Table S2: **Representation model comparison for different network sizes**. Time per iteration (in milliseconds), relative L_2 error (RL_2), and Wasserstein distance (W_d) on the validation dataset for cKAN and MLP models across different parameter counts $|\theta|$. The cKAN and MLP architectures were chosen to match parameter counts approximately, ensuring a fair comparison. All models were trained using the velocity-vorticity formulation (VV), sequential training, and RBA-R.

Model	$\lvert\theta\rvert(10^3)$	$t(ms/it)$	$RL_2 : \{u$	v	w	$T\}(\%)$	$W_d : \{u$	v	w	$T\}(10^{-3})$
MLP	50.5	112	19.4	26.0	20.3	7.42	1.32	1.59	1.05	16.0
cKAN	52.2	**92.3**	13.6	16.2	14.6	5.38	1.11	0.73	0.56	16.7
MLP	99.9	196	14.0	16.5	14.8	4.46	1.13	0.76	0.45	6.80
cKAN	101	160	10.6	12.0	12.6	3.64	0.71	0.48	0.45	4.96
MLP	149	280	11.3	13.0	13.0	4.13	0.83	0.45	0.36	13.0
cKAN	151	223	9.83	11.2	12.0	**3.45**	0.67	0.68	0.28	**4.38**
MLP	281	339	10.1	11.9	12.4	3.50	0.69	0.38	0.32	6.09
cKAN	279	359	**8.72**	**9.80**	**11.6**	4.06	**0.56**	**0.32**	**0.29**	14.5

points used in our model ($N_c = 820,000$). However, a direct numerical comparison between DNS and PIML is misleading, as collocation points in PIML are defined in 4D space-time, whereas DNS resolution refers to a fixed spatial grid at each time step. Consequently, the number of collocation points per snapshot is approximately two orders of magnitude smaller than the equivalent DNS grid resolution.

Accuracy To provide a deeper comparison of model accuracy, we evaluate performance using two key metrics: relative L_2 (RL_2) error, which provides an overall measure of convergence, and Wasserstein distance (Wd), which captures differences in distribution, particularly useful for assessing whether the model accurately reconstructs the tails, which may be related to fine-scale turbulent structures.

Table S2 and Figure S3 (A) and (B) show that the best-performing model for inferring hidden temperature fields is cKAN with 151k parameters, achieving an RL_2 of 3.45% and a Wd of 4.38×10^{-3}. Notably, an MLP with 282k parameters achieves a comparable performance with RL_2 = 3.50% and $Wd = 6.09 \times 10^{-3}$. However, cKAN with 279k parameters exhibits a slight performance

degradation, reaching $RL_2 = 4.06\%$ and $Wd = 14.5 \times 10^{-3}$.

For velocity reconstruction, the best-performing model is cKAN with 282k parameters, achieving RL_2 errors of 8.72% for u, 9.80% for v, and 11.6% for w. These trends can also be observed in Figure S3 (C), which illustrates the RL_2 convergence history across different models. Notably, the lowest Temperature RL_2 value is attained by the cKAN model with 279k parameters, achieving $RL_2 = 3.37\%$. However, due to the increased network complexity, the training process introduces oscillations that cause the final accuracy to deteriorate. This suggests that while increasing expressivity in cKAN models can initially improve performance, stability issues emerge at higher parameter counts.

Particularly, cKAN demonstrates relatively strong performance even at lower parameter counts, whereas MLP performance deteriorates significantly in this regime. As shown in Table S2 and Figure S3 (A) and (B), cKAN models with lower parameter counts maintain consistent accuracy, while MLPs struggle and exhibit a notable drop in performance. This indicates that cKANs are more parameter-efficient, achieving competitive results even with fewer parameters, making them particularly suitable for applications where network size is constrained.

On the other hand, as shown in Figure S3 (C), MLP with 282k parameters outperforms all cKAN models before switching to the L_1 loss, highlighting that for certain stages of training, traditional MLP architectures may exhibit faster initial convergence. However, after the transition, cKAN models recover more effectively, suggesting that they are more adaptable to sequential training. This observation aligns with (*83*), which suggests that B-spline KANs exhibit greater resilience to catastrophic forgetting. These results collectively demonstrate that cKAN achieves strong predictive performance with fewer parameters compared to MLPs, making it a competitive alternative for temperature field inference.

Computational Time While B-Spline KANs perform relatively well with fewer parameters, their training times remain significantly higher, as reported in (*83*).To address these limitations, several KAN variants have been introduced (*98–102*). In particular, (*31*) proposed cKANs and compared them with B-Spline KANs and MLPs. Their results showed that cKANs are significantly faster than B-Spline KANs while maintaining competitive accuracy. However, when training with full-batch updates, cKANs were slower than MLPs, with this difference increasing for larger networks.

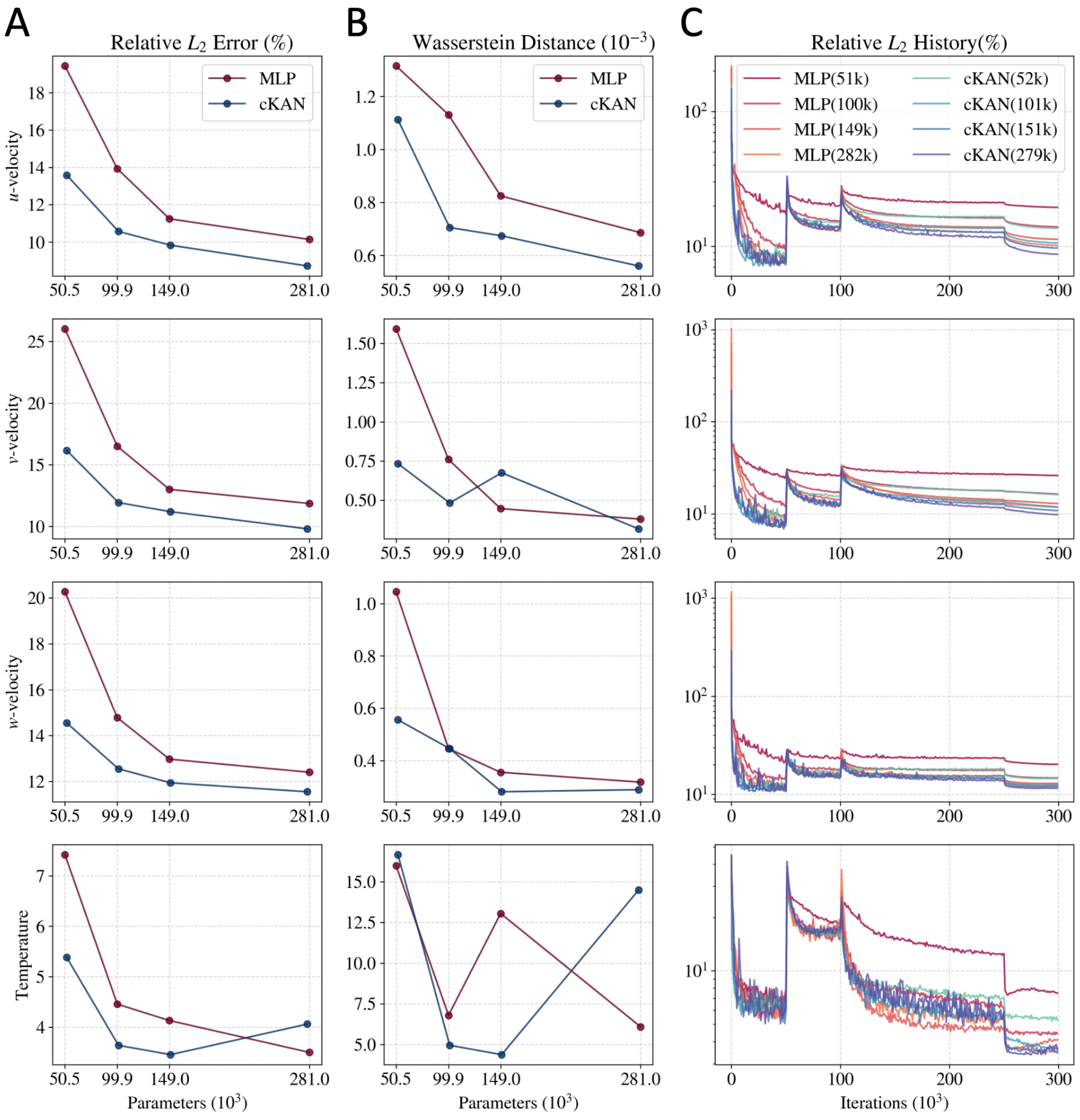

Figure S3: **Representation model comparison for different network sizes**. (A) Temperature relative L_2 error (RL_2) and (B) Wasserstein distance (W_d) on the validation dataset for cKAN and MLP models across different parameter counts $|\theta|$. The cKAN model achieves the best final performance with 151k parameters (RL_2 = 3.45%), while MLP performance degrades at smaller network sizes. (C) Temperature RL_2 convergence history. CKAN attains the lowest RL_2 with 279k parameters (RL_2 = 3.37%), but increasing complexity introduces oscillations, affecting final accuracy. Before switching to the L_1 loss, the MLP with 282K parameters outperforms all cKAN models, demonstrating cKAN's adaptability to training transitions.

Subsequent studies (*61*) demonstrated that this gap could be reduced by using smaller batch sizes, making cKANs as fast as MLPs for smaller networks and batch sizes.

In this study, we conducted a scalability analysis comparing MLPs and cKANs across different network sizes to validate these observations further. Table S2 confirms this pattern: for lower parameter counts ($\sim 50k$) with a small batch size ($bs = 5,000$), cKANs outperform MLPs, achieving a per-iteration time of 92.3ms compared to 112ms for MLPs. This advantage persists up to 150k parameters, where cKANs remain competitive. However, for larger networks (280k parameters), cKANs become slightly slower than MLPs.

This slowdown could be primarily caused by increased computational graph complexity in larger networks and memory inefficiencies in Chebyshev layer operations. Each Chebyshev layer processes large tensors of shape (batch size, polynomial order, number of inputs, number of outputs), leading to higher memory overhead and reduced efficiency at scale.

These findings indicate that cKANs remain a viable choice for PIML applications, where moderate network sizes are typically sufficient. However, for significantly larger architectures, MLPs may be preferable due to their more scalable computational structure.

Loss Landscapes analysis The original KAN model (B-Spline KAN) empirically exhibited performance degradation at high parameter counts, raising concerns about its suitability for real-world tasks (*103*). To address these limitations, several variations of KANs have been introduced (*98–102*). Notable examples include *FastKANs* (*98*), which utilize radial basis functions (RBFs), and *chebyKANs* (*101*), which leverage Chebyshev polynomials as basis functions. These approaches improve computational efficiency but still exhibit challenges.

For instance, *chebyKANs* employing a geometric representation (i.e., defining polynomials as $T_n(\theta) = \cos(n\theta)$) are computationally faster than B-spline KANs but suffer from instabilities. Specifically, as pointed out in (*31*), chebyKANs loss landscape contains discontinuities due to undefined values arising from the transformation $\theta = \arccos(x)$, which is restricted to $\arccos : \mathbb{R} \to (-1, 1)$. However, (*31*) empirically demonstrated that these instabilities stem from numerical precision issues in single-precision computations. They further showed that this issue could be mitigated by using the recursive Chebyshev formulation ($T_n(x) = 2xT_{n-1}(x) - T_{n-2}(x)$), which avoids the explicit computation of the inverse cosine function. The authors showed that this reformulation,

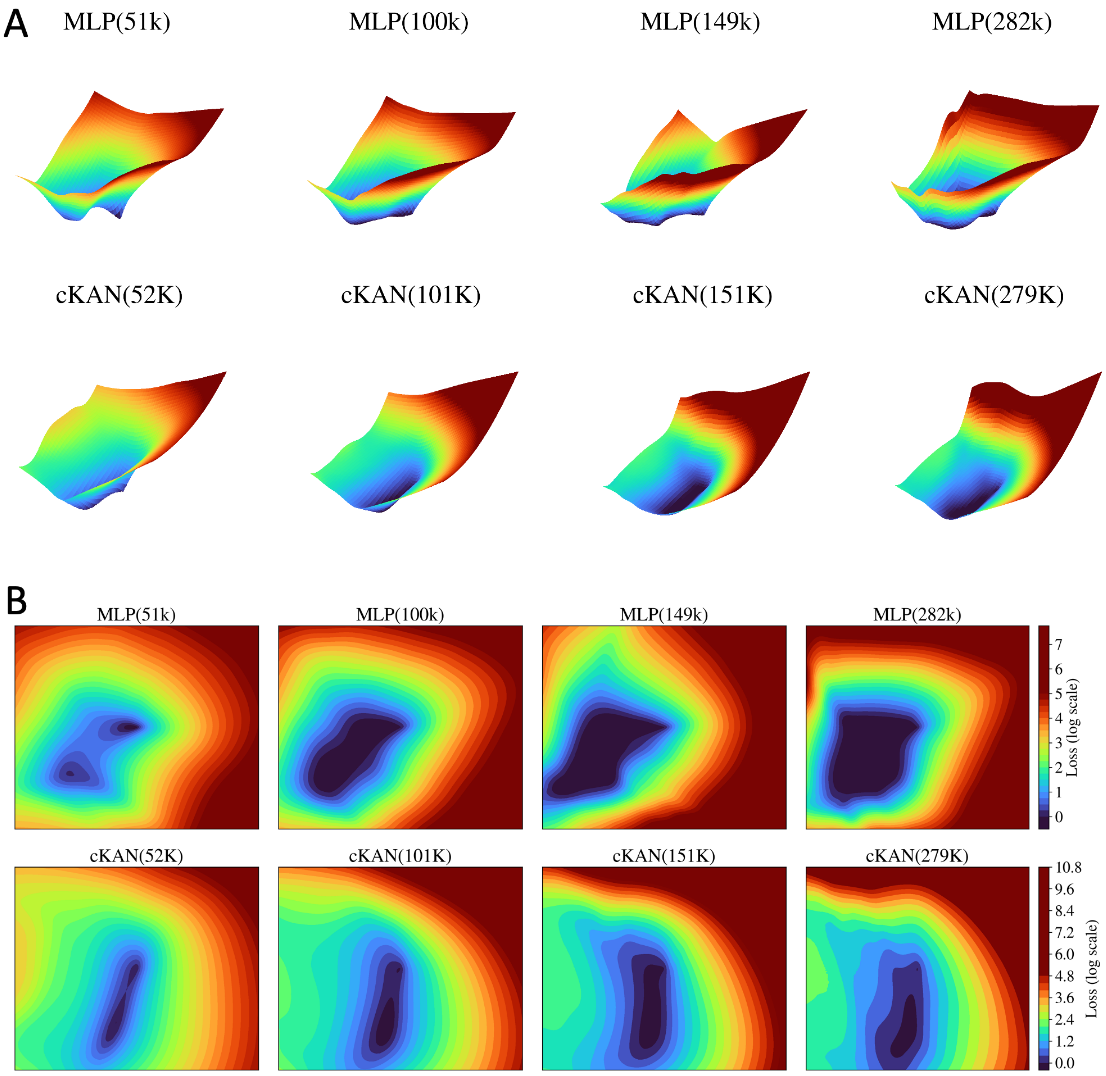

Figure S4: Comparison of loss landscapes for MLP and cKAN architectures under the full model setup (i.e., RBA-R sequential training and velocity-vorticity formulation) across different parameter counts. (A) 3D representation of the loss landscape for MLP (first row) and cKAN (second row) models. (B) Contour plot of the loss landscape for MLP (first row) and cKAN (second row) models. The results indicate that cKAN exhibits a smoother loss landscape, particularly for lower parameter counts, whereas MLPs in this regime tend to develop multiple local minima. However, the contour plots reveal that as the parameter count increases, the cKAN loss landscape becomes more slightly rugged, which may contribute to performance degradation.

referred to in this study as cKAN, restores model stability and induces loss landscapes comparable to MLPs.

To further examine this claim, we visualize the loss landscape of the full model (i.e., RBA-R sequential training and the velocity-vorticity formulation) for both MLPs and cKANs. To obtain these plots, we follow (*31, 39, 104, 105*) and reformulate the objective loss function ($\mathcal{L}$) as described in equation S36.

$$f(x_1, x_2) = \mathcal{L}(\theta_n + x_1\varepsilon_1 + x_2\varepsilon_2), \tag{S36}$$

here ε_1 and ε_2 are the direction vectors corresponding to the first two components obtained by Principal Component Analysis of the matrix $[\theta_1 - \theta_n, \theta_2 - \theta_n, ..., \theta_{n-1} - \theta_n]$, where θ are the trainable parameters, n is a subscript that denotes the nth iteration and x_1, x_2 are the directional weights associated to ε_1 and ε_2.

Figures S4(A) and (B) present 3D representations of the loss landscape and corresponding contour plots with varying parameter counts for MLP and cKAN, respectively. These figures suggest that both models exhibit comparable landscapes, with cKAN potentially inducing a smoother loss surface, particularly at lower parameter counts, where MLPs develop multiple local minima (see Figure S4(B)). Additionally, the contour plots reveal that as the parameter count increases, the cKAN loss landscape becomes more rugged, which may contribute to the slight performance degradation observed in the results.

The results suggest that while KAN-based models may present optimization challenges at higher parameter counts, structured modifications such as cKAN can mitigate these issues and provide a stable alternative. In this example, cKAN exhibited better performance and stability compared to MLPs. However, in their current state, cKANs may not scale as effectively as MLPs for very large networks. Despite this, for PIML applications, they remain a viable choice. Notably, cKAN demonstrated strong performance with approximately the same parameter count (279k; 8 layers, 81 neurons per layer, and polynomial order 5) as an MLP architecture commonly used in previous AIVst studies (*59, 60, 82*) (282k; 8 layers, 200 neurons per layer).

Ablation Study

The proposed AIVT method comprises four key components that enhance the baseline AIV model: (1) using cKAN as a representation model instead of MLP, (2) reformulating the PDE into the

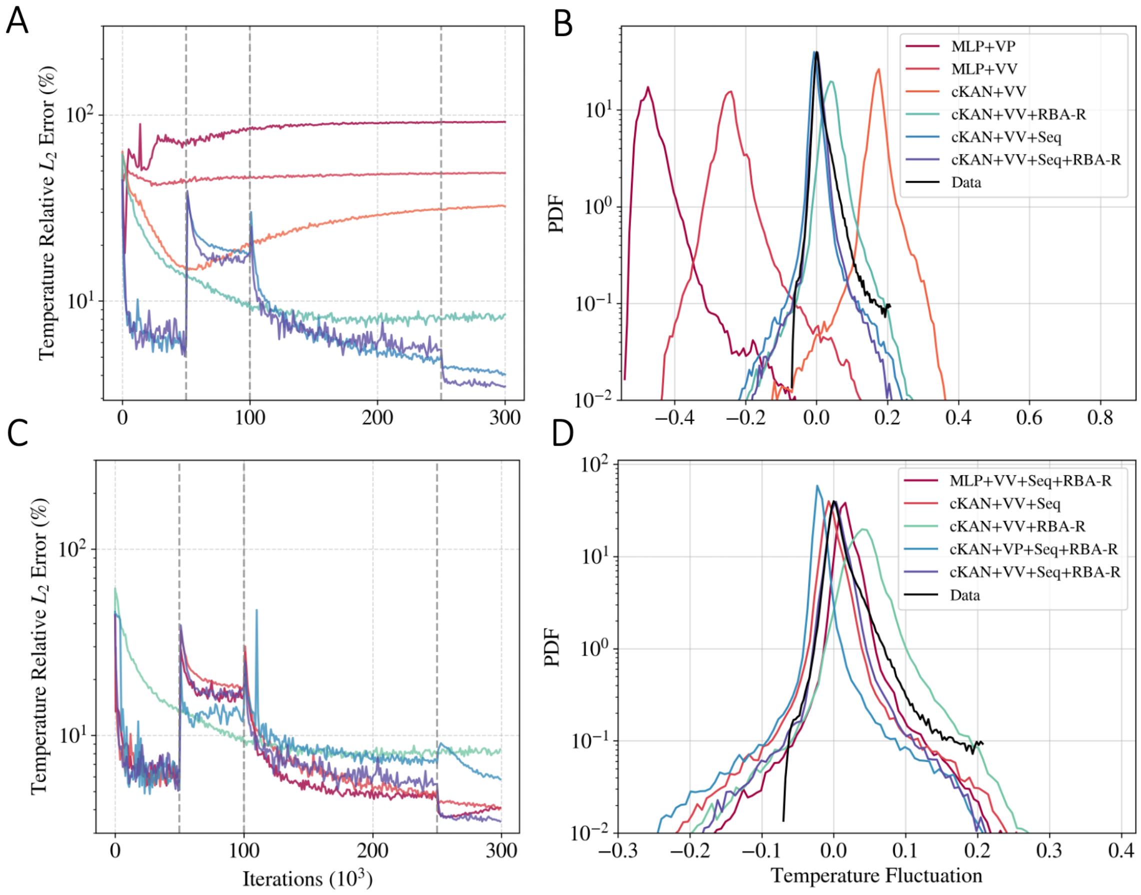

Figure S5: **Ablation study**. (A) Temperature relative L_2 error convergence history and (B) final temperature probability density function (PDF), comparing the baseline model (MLP+VP) with sequential variations of the proposed framework, including cKAN+VV, cKAN+VV+Seq, cKAN+VV+RBA-R, and cKAN+VV+Seq+RBA-R. The results indicate that each modification progressively enhances model performance in both temperature RL_2 and distribution alignment. Additionally, they highlight that sequential training and RBA-R independently contribute to improved convergence in the reference model (cKAN+VV). (C) Temperature relative L_2 error convergence history and (D) final temperature PDF for a single-component ablation study. The model without sequential training performs worse than the others, confirming sequential training as the most critical enhancement for reducing error and aligning the predicted distribution with experimental data. However, RBA-R plays a key role in accelerating convergence during training and achieving optimal performance in both distribution alignment and temperature RL_2 error.

Table S3: **Ablation study**. Time per iteration (in milliseconds), relative L_2 error (RL_2), and Wasserstein distance (W_d) comparison on the validation dataset. (A) Comparison between the baseline model (MLP+VP) and its subsequent enhancements: incorporating the velocity-vorticity formulation (MLP+VV), changing the representation model (cKAN+VV), further improving it with sequential training (cKAN+VV+Seq), and finally enhancing it with RBA-R (cKAN+VV+Seq+RBA-R). While the baseline model achieves better data-driven velocity reconstruction, it performs significantly worse in hidden temperature inference. Each modification further improves performance, with the VV formulation and sequential training providing the most significant improvements. (B) Single-component ablation study. Sequential training is the most critical enhancement, followed by the velocity-vorticity (VV) formulation. Notably, the fully enhanced MLP model achieves comparable RL_2 to the fully enhanced cKAN model; however, it exhibits greater distributional discrepancies W_d and higher computational time. Finally, while the benefits of RBA-R are less pronounced when sequential training is included, its contribution is still significant. Specifically, adding RBA-R reduces the error of the reference model (cKAN+VV) from 32.3% to 8.49%. All networks in this analysis have approximately $|\theta| \approx 150k$ trainable parameters.

	Model	$t(ms/it)$	$RL_2 : \{u$	v	w	$T\}(\%)$	$W_d : \{u$	v	w	$T\}(10^{-3})$
A	MLP+VP(Baseline)	181	**4.66**	**3.91**	**9.03**	91.5	**0.10**	**0.04**	**0.14**	475
	MLP+VV	284	7.36	9.39	10.8	48.7	0.43	0.29	0.26	251
	cKAN+VV	223	6.34	7.51	10.3	32.3	0.35	0.27	0.29	166
	cKAN+VV+Seq	223	9.63	12.7	11.8	4.0	0.60	0.75	0.33	9.94
	**cKAN+VV+Seq+RBA-R**	223	9.83	11.2	11.9	**3.45**	0.67	0.68	0.28	**4.38**
B	MLP+VV+Seq+RBA-R	284	11.3	13.0	13.0	4.13	0.83	0.45	0.36	13.1
	cKAN+VV+RBA-R	225	9.35	8.03	11.8	8.49	0.77	0.24	0.22	35.0
	cKAN+VV+Seq	223	9.63	12.7	11.8	4.0	0.60	0.75	0.33	9.94
	cKAN+VP+Seq+RBA-R	**158**	5.54	4.0	9.0	5.98	0.15	0.13	0.19	25.6

velocity-vorticity (VV) formulation, and two optimization algorithm improvements: (3) residual-based attention with resampling (RBA-R) and (4) sequential training (Seq). Based on this structure, our method can be represented as a composition of these subcomponents, i.e., AIVT = cKAN + VV + RBA-R + Seq. In contrast, the baseline AIV models (*59, 60*) utilize an MLP and the velocity-pressure (VP) formulation (i.e., MLP+VP).

To further analyze and interpret the impact of each component, we conduct two ablation studies. First, we evaluate model performance by progressively incorporating enhancements, starting from the baseline (MLP+VP), then adding the velocity-vorticity formulation (MLP+VV), replacing MLP with cKAN (cKAN+VV), introducing sequential training (cKAN+VV+Seq), and finally incorporating RBA-R (cKAN+VV+Seq+RBA-R). As shown in Table S3(A), while the baseline model achieves better data-driven velocity reconstruction, it performs significantly worse in hidden temperature inference. Each modification further improves performance, with the VV formulation and sequential training contributing the most substantial gains. Figure S5(A) and (B) demonstrate that each enhancement improves model convergence and aligns the predicted temperature distribution more closely with experimental data. Additionally, Figure S5(A) indicates that both RBA-R and sequential training independently enhance the reference model's (cKAN+VV) performance.

Second, we perform a one-component ablation study by removing one component at a time and evaluating the model performance of the ablated system. As shown in Table S3 (B) and Figure S5 (C) and (D), sequential training is the most critical enhancement in our approach. It simplifies the optimization problem by dividing the learning process into substeps and guiding temperature reconstruction using the theoretical average temperature profile.

Interestingly, the fully optimized model without the VV formulation, i.e., cKAN+VP+Seq+RBA-R, outperforms the full model in velocity reconstruction while being significantly faster. However, the Wasserstein distance (W_d) reveals a key trade-off: while the RL_2 error remains comparable, the W_d for this model is significantly larger, indicating that VV plays a crucial role in improving distributional alignment. This suggests that, although the VP formulation efficiently achieves low RL_2 errors, it struggles to capture the full distribution, as illustrated in Figure S5(D).

To further investigate this pattern, we analyze the loss landscapes of the different ablated systems in Figure S6. Notably, VP-based models exhibit a more rugged loss landscape with multiple local minima, confirming that simultaneously inferring both pressure and temperature increases

optimization complexity. In contrast, VV-based models tend to produce a smoother loss surface, which may explain their improved alignment with the experimental data distribution. However, as shown in Table S3, the vorticity formulation comes at a significantly higher computational cost. Similarly, while the MLP+VV+Seq+RBA-R model achieves comparable RL_2 performance to the full model, its W_d is nearly four times larger, indicating a significant distribution mismatch.

Finally, while the impact of RBA-R is less pronounced when sequential training is included, its contribution remains significant. Specifically, adding RBA-R reduces the error of the reference model (cKAN+VV) from 32.3% to 8.49% (see Table S3). This effect is further demonstrated in Figure S5(A), where RBA-R substantially enhances convergence in the reference model (cKAN+VV) and accelerates training, particularly in the final stage (Figure S5(C)).

Additionally, Figure S5(D) highlights that RBA-R plays a crucial role in improving distributional alignment, ensuring that the predicted temperature distribution closely matches the experimental data. In summary, both Sequential Training and RBA-R significantly enhance model performance. However, if one of them is removed, the model remains robust, suggesting that their contributions partially compensate for each other. This does not imply that either component is non-essential; rather, their individual effects may be masked by the presence of the other.

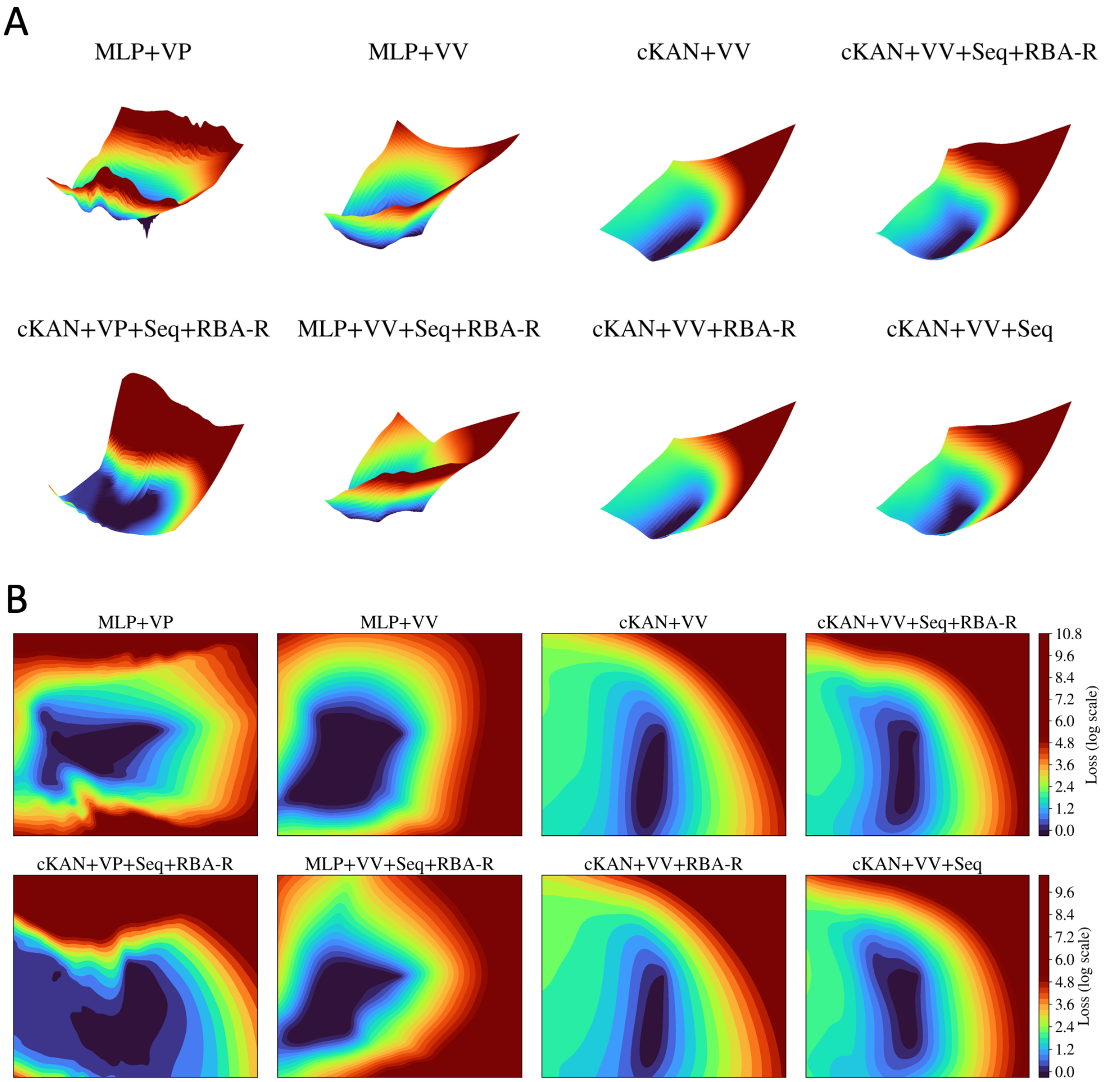

Figure S6: Comparison of loss landscapes for MLP and cKAN architectures under the ablation study setup, analyzing the impact of model enhancements. (A) 3D representation of the loss landscape for MLP (first row) and cKAN (second row) models. (B) Contour plot of the loss landscape for MLP (first row) and cKAN (second row) models. The results indicate that both representation models exhibit comparable landscapes. However, models using the velocity-pressure (VP) formulation display a more rugged landscape with multiple local minima, suggesting that the increased complexity of simultaneously learning both the pressure and temperature fields significantly impacts optimization stability.

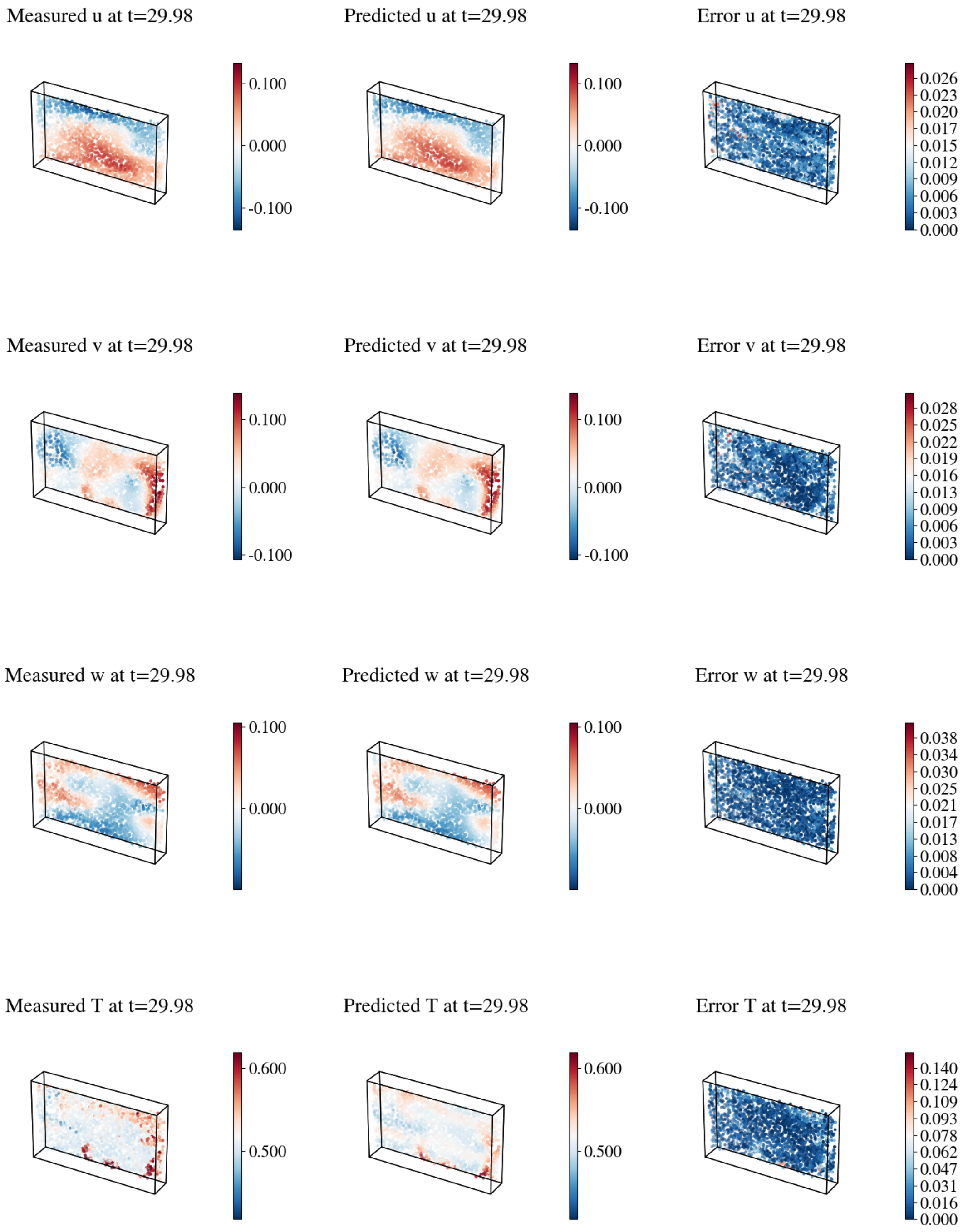

Figure S7: Measured and predicted flow fields and their corresponding absolute error for a representative time step (worst case). (First row) Velocity in x direction. (Second row) Velocity in y direction. (Third row) Velocity in z direction. (Fourth row) Temperature.

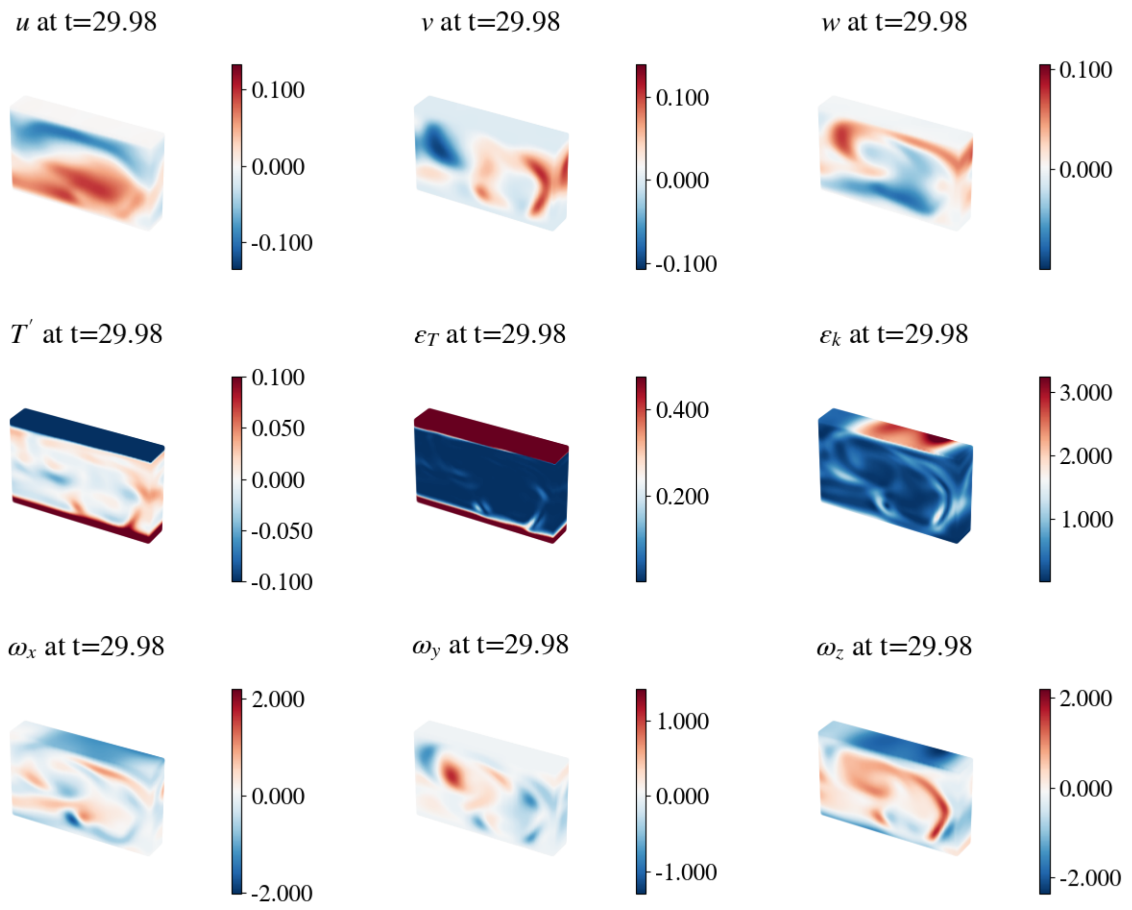

Figure S8: Reconstructed 3D velocities and inferred flow fields at a representative time step. (First row) Velocity components in the x, y and z directions. (Second row) temperature fluctuations, thermal and viscous dissipation. (Third row) Vorticity components in the x, y and z directions.

Table S4: **Pretraining stages: Global Weights (Stage 1 and 2)**. In the first "data-driven" stage, the model follows a purely empirical approach, learning the velocity field from available measurements and the theoretical temperature profile. In the second "partial physics" stage, the contribution of the theoretical profile is significantly reduced, and the PDE constraints are activated using a lower Rayleigh number (Ra).

Stage	Loss Term	Sub-term	Global Weight (m_α)
1	Data	u	1000
1	Data	v, w	10000
1	Data	T^*	10
1	Boundaries	u, v, w, w_y	10
1	Equations	Momentum x, z	10^{-10}
1	Equations	Momentum y	10^{-10}
1	Equations	Temperature T	10^{-10}
1	Equations	Conservation of mass	1
1	Equations	Vector Identities x, y, z	1
2	Data	u	100
2	Data	v, w	1000
2	Data	T^*	0.1
2	Boundaries	u, v, w, w_y	10
2	Equations	Momentum x, z	1
2	Equations	Momentum y	10
2	Equations	Temperature T	10
2	Equations	Conservation of mass	1
2	Equations	Vector Identities x, y, z	1

Table S5: **Global Weights (Stage 3 and 4)**. After pretraining, the contribution of the theoretical profile is further reduced, and the model is trained using the true Rayleigh number (Ra) with an L_2 norm loss. In the final stage, the loss function is switched to the L_1 norm.

Stage	Loss Term	Sub-term	Global Weight (m_α)
3	Data	u	10
3	Data	v, w	100
3	Data	T^*	0.01
3	Boundaries	u, v, w, w_y	10
3	Equations	Momentum x, z	1
3	Equations	Momentum y	10
3	Equations	Temperature T	10
3	Equations	Conservation of mass	1
3	Equations	Vector Identities x, y, z	1
4	Data	u	1
4	Data	v, w	10
4	Data	T^*	0.001
4	Boundaries	u, v, w, w_y	1
4	Equations	Momentum x, z	0.1
4	Equations	Momentum y	1
4	Equations	Temperature T	1
4	Equations	Conservation of mass	0.1
4	Equations	Vector Identities x, y, z	0.1

Caption for Movie S1. Measured and predicted flow fields and their corresponding error T(First row) Velocity in x direction. (Second row) Velocity in y direction. (Third row) Velocity in z direction. (Fourth row) Temperature.

Caption for Movie S2. Reconstructed and inferred 3D flow fields (First row) velocity magnitude, temperature, vorticity magnitude, (Second row) convective heat transfer, and thermal and viscous dissipation rates.

Caption for Movie S3. Reconstructed and inferred 3D flow fields components (First row) Velocity components in the x, y and z directions. (Second row)temperature fluctuations, thermal and viscous dissipation. (Third row)Vorticity components in the x, y and z directions.

REFERENCES AND NOTES

1. C. Guervilly, P. Cardin, N. Schaeffer, Turbulent convective length scale in planetary cores. *Nature* **570**, 368–371 (2019).

2. B. E. Mapes, R. A. Houze Jr, Cloud clusters and superclusters over the oceanic warm pool. *Mon. Weather Rev.* **121**, 1398–1416 (1993).

3. J. Schumacher, K. R. Sreenivasan, *Colloquium*: Unusual dynamics of convection in the Sun. *Rev. Mod. Phys.* **92**, 041001 (2020).

4. R. M. B. Young, P. L. Read, Forward and inverse kinetic energy cascades in Jupiter's turbulent weather layer. *Nat. Phys.* **13**, 1135–1140 (2017).

5. I. Bitharas, N. Parab, C. Zhao, T. Sun, A. D. Rollett, A. J. Moore, The interplay between vapour, liquid, and solid phases in laser powder bed fusion. *Nat. Commun.* **13**, 2959 (2022).

6. H. Otto, C. Naumann, C. Odenthal, C. Cierpka, Unsteady inherent convective mixing in thermal-energy-storage systems during standby periods. *PRX Energy* **2**, 043001 (2023).

7. C. M. S. Martin, J. K. Lundquist, A. Clifton, G. S. Poulos, S. J. Schreck, Wind turbine power production and annual energy production depend on atmospheric stability and turbulence. *Wind Energy Sci.* **1**, 221–236 (2016).

8. P. Milan, M. Wächter, J. Peinke, Turbulent character of wind energy. *Phys. Rev. Lett.* **110**, 138701 (2013).

9. H. Bénard, Les tourbillons cellulaires dans une nappe liquide transportant de la chaleur par convection en régime permanent. *Ann. Chim. Phys.* **23**, 62–144 (1901).

10. L. Rayleigh, On convection currents in a horizontal layer of fluid, when the higher temperature is on the under side. *Phil. Mag.* **32**, 529–546 (1916).

11. P. Manneville, “Rayleigh-Bénard convection: Thirty years of experimental, theoretical, and modeling Work” in *Dynamics of Spatio-Temporal Cellular Structures* (Springer, 2006), pp. 41–65.

12. G. Ahlers, S. Grossmann, D. Lohse, Heat transfer and large scale dynamics in turbulent Rayleigh-Bénard convection. *Rev. Mod. Phys.* **81**, 503–537 (2009).

13. F. Chillà, J. Schumacher, New perspectives in turbulent Rayleigh-Bénard convection. *Eur. Phys. J. E* **35**, 58 (2012).

14. K. P. Iyer, J. D. Scheel, J. Schumacher, K. R. Sreenivasan, Classical 1/3 scaling of convection holds up to Ra = 10^{15}. *Proc. Natl. Acad. Sci. U.S.A.* **117**, 7594–7598 (2020).

15. N. Jansson, M. Karp, A. Perez, T. Mukha, Y. Ju, J. Liu, S. Pall, E. Laure, T. Weinkauf, J. Schumacher, P. Schlatter, S. Markidis, “Exploring the ultimate regime of turbulent Rayleigh-Bénard convection through unprecedented spectral-element simulations” in *Proceedings of the International Conference for High Performance Computing, Networking, Storage and Analysis* (ACM, 2023), pp. 1–9.

16. M. Raffel, C. E. Willert, F. Scarano, C. J. Kähler, S. T. Wereley, J. Kompenhans, *Particle Image Velocimetry: A practical guide* (Springer International Publishing, 2018).

17. C. J. Kähler, T. Astarita, P. P. Vlachos, J. Sakakibara, R. Hain, S. Discetti, R. la Foy, C. Cierpka, Main results of the 4th International PIV Challenge. *Exp. Fluids* **57**, 97 (2016).

18. Z. Deng, J. König, C. Cierpka, A combined velocity and temperature measurement with an LED and a low-speed camera. *Meas. Sci. Technol.* **33**, 115301 (2022).

19. J. Massing, C. J. Kähler, C. Cierpka, A volumetric temperature and velocity measurement technique for microfluidics based on luminescence lifetime imaging. *Exp. Fluids* **59**, 163 (2018).

20. R. Segura, M. Rossi, C. Cierpka, C. J. Kähler, Simultaneous three-dimensional temperature and velocity field measurements using astigmatic imaging of non-encapsulated thermo-liquid crystal (TLC) particles. *Lab Chip* **15**, 660–663 (2015).

21. M. Stelter, F. J. W. A. Martins, F. Beyrau, B. Fond, Thermographic 3D particle tracking velocimetry for turbulent gas flows. *Meas. Sci. Technol.* **34**, 074008 (2023).

22. M. Rietz, O. Garbrecht, W. Rohlfs, R. Kneer, "Combined three-dimensional flow-and temperature-field measurement using digital light field photography" in *International Heat Transfer Conference Digital Library* (Begel House Inc., 2014).

23. D. Schiepel, D. Schmeling, C. Wagner, Simultaneous tomographic particle image velocimetry and thermometry of turbulent Rayleigh-Bénard convection. *Meas. Sci. Technol.* **32**, 095201 (2021).

24. T. Käufer, C. Cierpka, Volumetric Lagrangian temperature and velocity measurements with thermochromic liquid crystals. *Meas. Sci. Technol.* **35**, 035301 (2024).

25. D. Noto, H. N. Ulloa, J. A. Letelier, Reconstructing temperature fields for thermally-driven flows under quasi-steady state. *Exp. Fluids* **64**, 74 (2023).

26. C. Bauer, D. Schiepel, C. Wagner, Assimilation and extension of particle image velocimetry data of turbulent Rayleigh-Bénard convection using direct numerical simulations. *Exp. Fluids* **63**, 22 (2022).

27. P. Teutsch, T. Käufer, P. Mäder, C. Cierpka, Data-driven estimation of scalar quantities from planar velocity measurements by deep learning applied to temperature in thermal convection. *Exp. Fluids* **64**, 191 (2023).

28. E. Fonda, A. Pandey, J. Schumacher, K. R. Sreenivasan, Deep learning in turbulent convection networks. *Proc. Natl. Acad. Sci. U.S.A.* **116**, 8667–8672 (2019).

29. M. Kang, B. Kwon, Deep learning of forced convection heat transfer. *J. Heat Transfer* **144**, 021801 (2022).

30. M. Raissi, P. Perdikaris, G. E. Karniadakis, Physics-informed neural networks: A deep learning framework for solving forward and inverse problems involving nonlinear partial differential equations. *J. Comput. Phys.* **378**, 686–707 (2019).

31. K. Shukla, J. D. Toscano, Z. Wang, Z. Zou, G. E. Karniadakis, A comprehensive and FAIR comparison between MLP and KAN representations for differential equations and operator networks. arXiv:2406.02917 [cs.LG] (2024).

32. S. Wang, H. Wang, P. Perdikaris, On the eigenvector bias of Fourier feature networks: From regression to solving multi-scale PDEs with physics-informed neural networks. *Comput. Methods Appl. Mech. Eng.* **384**, 113938 (2021).

33. N. Sukumar, A. Srivastava, Exact imposition of boundary conditions with distance functions in physics-informed deep neural networks. *Comput. Methods Appl. Mech. Eng.* **389**, 114333 (2022).

34. T. Salimans, D. P. Kingma, Weight normalization: A simple reparameterization to accelerate training of deep neural networks. *Adv. Neural Inf. Process. Syst.* **29**, 901–909 (2016).

35. S. Wang, Y. Teng, P. Perdikaris, Understanding and mitigating gradient flow pathologies in physics-informed neural networks. *SIAM J. Sci. Comput.* **43**, A3055–A3081 (2021).

36. S. Wang, B. Li, Y. Chen, P. Perdikaris, PirateNets: Physics-informed Deep Learning with Residual Adaptive Networks. arXiv:2402.00326 [cs.LG] (2024).

37. Q. Zhang, C. Wu, A. Kahana, Y. Kim, Y. Li, G. E. Karniadakis, P. Panda, Artificial to spiking neural networks conversion for scientific machine learning. arXiv:2308.16372 [cs.NE] (2023).

38. X. Jin, S. Cai, H. Li, G. E. Karniadakis, NSFnets (Navier-Stokes flow nets): Physics-informed neural networks for the incompressible Navier-Stokes equations. *J. Comput. Phys.* **426**, 109951 (2021).

39. Z. Wang, X. Meng, X. Jiang, H. Xiang, G. E. Karniadakis, Solution multiplicity and effects of data and eddy viscosity on Navier-Stokes solutions inferred by physics-informed neural networks. arXiv:2309.06010 [physics.flu-dyn] (2023).

40. S. Basir, Investigating and Mitigating Failure Modes in Physics-informed Neural Networks (PINNs). arXiv:2209.09988 [cs.LG] (2022).

41. S. J. Anagnostopoulos, J. D. Toscano, N. Stergiopulos, G. E. Karniadakis, Residual-based attention in physics-informed neural networks. *Comput. Methods Appl. Mech. Eng.* **421**, 116805 (2024).

42. S. Wang, S. Sankaran, P. Perdikaris, Respecting causality for training physics-informed neural networks. *Comput. Methods Appl. Mech. Eng.* **421**, 116813 (2024).

43. L. D. McClenny, U. M. Braga-Neto, Self-adaptive physics-informed neural networks. *J. Comput. Phys.* **474**, 111722 (2023).

44. L. Lu, X. Meng, Z. Mao, G. E. Karniadakis, DeepXDE: A deep learning library for solving differential equations. *SIAM Rev.* **63**, 208–228 (2021).

45. C. Wu, M. Zhu, Q. Tan, Y. Kartha, L. Lu, A comprehensive study of non-adaptive and residual-based adaptive sampling for physics-informed neural networks. *Comput. Methods Appl. Mech. Eng.* **403**, 115671 (2023).

46. A. A. Howard, B. Jacob, S. H. Murphy, A. Heinlein, P. Stinis, Finite basis Kolmogorov-Arnold networks: Domain decomposition for data-driven and physics-informed problems. arXiv:2406.19662 [cs.LG] (2024).

47. W. Chen, A. A. Howard, P. Stinis, Self-adaptive weights based on balanced residual decay rate for physics-informed neural networks and deep operator networks. arXiv:2407.01613 [cs.LG] (2024).

48. S. Cai, Z. Wang, S. Wang, P. Perdikaris, G. E. Karniadakis, Physics-informed neural networks for heat transfer problems. *J. Heat Transfer* **143**, 060801 (2021).

49. V. Oommen, B. Srinivasan, Solving inverse heat transfer problems without surrogate models: A fast, data-sparse, physics informed neural network approach. *J. Comput. Inf. Sci. Eng.* **22**, 041012 (2022).

50. M. Mommert, R. Barta, C. Bauer, M.-C. Volk, C. Wagner, Periodically activated physics-informed neural networks for assimilation tasks for three-dimensional Rayleigh–Bénard convection. *Comput. Fluids* **283**, 106419 (2024).

51. P. Clark Di Leoni, L. Agasthya, M. Buzzicotti, L. Biferale, Reconstructing Rayleigh-Bénard flows out of temperature-only measurements using Physics-Informed Neural Networks. *Eur. Phys. J. E* **46**, 16 (2023).

52. V. Kag, K. Seshasayanan, V. Gopinath, Physics-informed data based neural networks for two-dimensional turbulence. *Phys. Fluids* **34**, 055130 (2022).

53. Y. Du, M. Wang, T. A. Zaki, State estimation in minimal turbulent channel flow: A comparative study of 4DVar and PINN. *Int. J. Heat Fluid Flow* **99**, 109073 (2023).

54. F. Pioch, J. H. Harmening, A. M. Müller, F.J. Peitzmann, D. Schramm, O. el Moctar, Turbulence modeling for physics-informed neural networks: Comparison of different RANS models for the backward-facing step flow. *Fluids* **8**, 43 (2023).

55. Y. Patel, V. Mons, O. Marquet, G. Rigas, Turbulence model augmented physics-informed neural networks for mean-flow reconstruction. *Phys. Rev. Fluids* **9**, 034605 (2024).

56. K. Zhou, S. J. Grauer, Flow reconstruction and particle characterization from inertial Lagrangian tracks. arXiv:2311.09076 [physics.flu-dyn] (2023).

57. M. Raissi, A. Yazdani, G. E. Karniadakis, Hidden fluid mechanics: Learning velocity and pressure fields from flow visualizations. *Science* **367**, 1026–1030 (2020).

58. S. Cai, Z. Wang, F. Fuest, Y. J. Jeon, C. Gray, G. E. Karniadakis, Flow over an espresso cup: Inferring 3-D velocity and pressure fields from tomographic background oriented Schlieren via physics-informed neural networks. *J. Fluid Mech.* **915**, A102 (2021).

59. K. A. Boster, S. Cai, A. Ladrón-de-Guevara, J. Sun, X. Zheng, T. du, J. H. Thomas, M. Nedergaard, G. E. Karniadakis, D. H. Kelley, Artificial intelligence velocimetry reveals in vivo

flow rates, pressure gradients, and shear stresses in murine perivascular flows. *Proc. Natl. Acad. Sci. U.S.A.* **120**, e2217744120 (2023).

60. S. Cai, H. Li, F. Zheng, F. Kong, M. Dao, G. E. Karniadakis, S. Suresh, Artificial intelligence velocimetry and microaneurysm-on-a-chip for three-dimensional analysis of blood flow in physiology and disease. *Proc. Natl. Acad. Sci. U.S.A.* **118**, e2100697118 (2021).

61. J. D. Toscano, L.-L. Wang, G. E. Karniadakis, KKANs: Kurkova-Kolmogorov-Arnold networks and their learning dynamics. arXiv:2412.16738 (2024).

62. D. Dabiri, Digital particle image thermometry/velocimetry: A review. *Exp. Fluids* **46**, 191–241 (2009).

63. A. Schröder, D. Schanz, 3D Lagrangian particle tracking in fluid mechanics. *Annu. Rev. Fluid Mech.* **55**, 511–540 (2023).

64. S. J. Anagnostopoulos, J. D. Toscano, N. Stergiopulos, G. E. Karniadakis, Learning in PINNs: Phase transition, total diffusion, and generalization. arXiv:2403.18494 (2024).

65. E. Brown, A. Nikolaenko, G. Ahlers, Reorientation of the large-scale circulation in turbulent Rayleigh-Bénard convection. *Phys. Rev. Lett.* **95**, 084503 (2005).

66. Y. Zhang, Y. X. Huang, N. Jiang, Y. L. Liu, Z. M. Lu, X. Qiu, Q. Zhou, Statistics of velocity and temperature fluctuations in two-dimensional Rayleigh-Bénard convection. *Phys. Rev. E* **96**, 023105 (2017).

67. J. D. Scheel, E. Kim, K. R. White, Thermal and viscous boundary layers in turbulent Rayleigh-Bénard convection. *J. Fluid Mech.* **711**, 281–305 (2012).

68. C. Sun, Y.-H. Cheung, K.-Q. Xia, Experimental studies of the viscous boundary layer properties in turbulent Rayleigh–Bénard convection. *J. Fluid Mech.* **605**, 79–113 (2008).

69. S. Moller, T. Käufer, A. Pandey, J. Schumacher, C. Cierpka, Combined particle image velocimetry and thermometry of turbulent superstructures in thermal convection. *J. Fluid Mech.* **945**, A22 (2022).

70. O. Shishkina, A. Thess, Mean temperature profiles in turbulent Rayleigh-Bénard convection of water. *J. Fluid Mech.* **633**, 449–460 (2009).

71. S. Horn, O. Shishkina, Rotating non-Oberbeck-Boussinesq Rayleigh-Bénard convection in water. *Phys. Fluids* **26**, 055111 (2014).

72. G. Tegze, F. Podmaniczky, Three-dimensional structure of the thermal boundary layer in turbulent Rayleigh-Bénard convection: A Lagrangian perspective. *Phys. Rev. Fluids* **9**, 074602 (2024).

73. R. J. Stevens, E. P. van der Poel, S. Grossmann, D. Lohse, The unifying theory of scaling in thermal convection: The updated prefactors. *J. Fluid Mech.* **730**, 295–308 (2013).

74. S.-L. Lui, K.-Q. Xia, Spatial structure of the thermal boundary layer in turbulent convection. *Phys. Rev. E* **57**, 5494–5503 (1998).

75. Q. Zhou, K.-Q. Xia, Thermal boundary layer structure in turbulent Rayleigh-Bénard convection in a rectangular cell. *J. Fluid Mech.* **721**, 199–224 (2013).

76. S. Grossmann, D. Lohse, Scaling in thermal convection: A unifying theory. *J. Fluid Mech.* **407**, 27–56 (2000).

77. P. L. Johnson, M. Wilczek, Multiscale velocity gradients in turbulence. *Annu. Rev. Fluid Mech.* **56**, 463–490 (2024).

78. C. Meneveau, Lagrangian dynamics and models of the velocity gradient tensor in turbulent flows. *Annu. Rev. Fluid Mech.* **43**, 219–245 (2011).

79. X. He, P. Tong, K.-Q. Xia, Measured thermal dissipation field in turbulent Rayleigh-Bénard convection. *Phys. Rev. Lett.* **98**, 144501 (2007).

80. F. Xu, L. Zhang, K.-Q. Xia, Experimental measurement of spatio-temporally resolved energy dissipation rate in turbulent Rayleigh-Bénard convection. *J. Fluid Mech.* **984**, A8 (2024).

81. M. Kaczorowski, K.-Q. Xia, Turbulent flow in the bulk of Rayleigh-Bénard convection: Small-scale properties in a cubic cell. *J. Fluid Mech.* **722**, 596–617 (2013).

82. J. D. Toscano, C. Wu, A. Ladrón-de-Guevara, T. du, M. Nedergaard, D. H. Kelley, G. E. Karniadakis, K. A. S. Boster, Inferring in vivo murine cerebrospinal fluid flow using artificial intelligence velocimetry with moving boundaries and uncertainty quantification. *Interface Focus* **14**, 20240030 (2024).

83. Z. Liu, Y. Wang, S. Vaidya, F. Ruehle, J. Halverson, M. Soljačić, T. Y. Hou, M. Tegmark, Kan: Kolmogorov-arnold networks. arXiv:2404.19756 (2024).

84. F. Girosi, T. Poggio, Representation properties of networks: Kolmogorov's theorem is irrelevant. *Neural Comput.* **1**, 465–469 (1989).

85. L. F. Guilhoto, P. Perdikaris, Deep learning alternatives of the Kolmogorov superposition theorem. arXiv:2410.01990 (2024).

86. V. Kurková, Kolmogorov's theorem and multilayer neural networks. *Neural Netw.* **5**, 501–506 (1992).

87. W. Cai, Z.-Q. J. Xu, Multi-scale deep neural networks for solving high dimensional PDEs. arXiv:1910.11710 (2019).

88. Z. Liu, W. Cai, Z.-Q. J. Xu, Multi-scale deep neural network (MscaleDNN) for solving Poisson-Boltzmann equation in complex domains. *Commun. Comput. Phys.* **28**, 1970–2001 (2020).

89. B. Wang, W. Zhang, W. Cai, Multi-scale deep neural network (MscaleDNN) methods for oscillatory stokes flows in complex domains. arXiv:2009.12729 (2020).

90. L. Liu, B. Wang, W. Cai, Linearized learning with multiscale deep neural networks for stationary Navier-Stokes equations with oscillatory solutions. *East Asian J. Appl. Math.* **13**, 740–758 (2022).

91. N. Ahmadi Daryakenari, M. De Florio, K. Shukla, G. E. Karniadakis, AI-Aristotle: A physics-informed framework for systems biology gray-box identification. *PLoS Comput. Biol.* **20**, e1011916 (2024).

92. Z. Zhang, T. Shen, Y. Zhang, W. Zhang, Q. Wang, AL-PKAN: A hybrid GRU-KAN network with augmented lagrangian function for solving PDEs (2024); https://ssrn.com/abstract=4957859.

93. E. C. Cyr, M. A. Gulian, R. G. Patel, M. Perego, N. A. Trask, "Robust training and initialization of deep neural networks: An adaptive basis viewpoint" in *Mathematical and Scientific Machine Learning* (PMLR, 2020), pp. 512–536.

94. A. D. Jagtap, K. Kawaguchi, G. E. Karniadakis, Adaptive activation functions accelerate convergence in deep and physics-informed neural networks. *J. Comput. Phys.* **404**, 109136 (2020).

95. A. D. Jagtap, K. Kawaguchi, G. Em Karniadakis, Locally adaptive activation functions with slope recovery for deep and physics-informed neural networks. *Proc. R. Soc. A: Math. Phys. Eng. Sci.* **476**, 20200334 (2020).

96. I. Loshchilov, F. Hutter, Fixing weight decay regularization in adam. arXiv:1711.05101 (2017).

97. J. D. Scheel, M. S. Emran, J. Schumacher, Resolving the fine-scale structure in turbulent Rayleigh–Bénard convection. *New J. Phys.* **15**, 113063 (2013).

98. Z. Li, Kolmogorov-Arnold networks are radial basis function networks. arXiv:2405.06721 (2024).

99. Z. Bozorgasl, H. Chen, Wav-KAN: Wavelet Kolmogorov-Arnold networks. arXiv:2405.12832 (2024).

100. NLNR, JacobiKAN (2024); https://github.com/mintisan/awesome-kan/.

101. S. Sidharth, A. Keerthana, R. Gokul, K. Anas, Chebyshev polynomial-based Kolmogorov-Arnold networks: An efficient architecture for nonlinear function approximation. arXiv:2405.07200 (2024).

102. S. S. Bhattacharjee, TorchKAN: Simplified KAN model with variations (2024); https://github.com/1ssb/torchkan/.

103. A. Pal, D. Das, “Understanding the limitations of B-Spline KANs: Convergence dynamics and computational efficiency” in *NeurIPS 2024 Workshop on Scientific Methods for Understanding Deep Learning* (NeurIPS, 2024).

104. H. Li, Z. Xu, G. Taylor, C. Studer, T. Goldstein, Visualizing the loss landscape of neural nets. *Adv. Neural Inf. Process. Syst.* **31**, 6799–6810 (2018).

105. A. Krishnapriyan, A. Gholami, S. Zhe, R. Kirby, M. W. Mahoney, Characterizing possible failure modes in physics-informed neural networks. *Adv. Neural Inf. Process. Syst.* **34**, 26548–26560 (2021).
